# Supplementary material for: Effect of vacuum freeze drying and hot air drying on dried mulberry fruit quality
Source: PLoS One. 2023 Jun 23;18(6):e0283303. doi: 10.1371/journal.pone.0283303 (PMC10289396; doi:10.1371/journal.pone.0283303)
Supplement: S1 File — (DOCX) [file pone.0283303.s001.docx]

amino acid content

|  | fresh fruit | vacuum freeze drying | hot air drying |
| --- | --- | --- | --- |
| D1 | 509.01±6.42 Aa | 493.02±6.42 Ab | 428.02±8.63 Bc |
| D2 | 433.48±2.86 Aa | 418.79±6.64 Ab | 388.58±6.94 Bc |
| D3 | 576.39±8.11 Aa | 556.49±5.77 Bb | 486.79±8.99 Bc |

soluble protein content

|  | fresh fruit | vacuum freeze drying | hot air drying |
| --- | --- | --- | --- |
| D1 | 59.92±1.66 Aa | 57.35±1.54 Aa | 38.57±1.14 Bb |
| D2 | 49.03±2.07 Aa | 45.65±2.26 Aa | 30.85±1.86 Bb |
| D3 | 70.74±2.32 Aa | 69.86±1.50 Aa | 55.41±1.26 Bb |

ascorbic acid content

|  | fresh fruit | vacuum freeze drying | hot air drying |
| --- | --- | --- | --- |
| D1 | 16.14±3.45 Aa | 14.67±0.48 Bb | 8.01±0.27 Cc |
| D2 | 82.03±2.07 Aa | 77.95±3.90 Ab | 56.42±2.58 Bc |
| D3 | 116.13±3.75 Aa | 101.26±0.98 Bb | 78.86±3.39 Cc |

soluble sugar content

|  | fresh fruit | vacuum freeze drying | hot air drying |
| --- | --- | --- | --- |
| D1 | 349.55±25.51 Aa | 343.50±4.45 Aa | 255.20±10.17 Bb |
| D2 | 439.86±1.18 Aa | 380.97±8.33 Bb | 278.31±16.19 Cc |
| D3 | 270.28±13.49 Aa | 231.35±4.19 Bb | 207.05±16.99 Bb |

polyphenol content

|  | fresh fruit | vacuum freeze drying | hot air drying |
| --- | --- | --- | --- |
| D1 | 15.46±0.93 Aa | 13.42±0.54 Ab | 8.27±0.54 Bc |
| D2 | 138.27±6.58 Aa | 116.49±6.42 Bb | 89.08±6.83 Cc |
| D3 | 165.01±1.79 Aa | 157.82±3.91 Bb | 144.00±4.08 Bc |

resveratrol content

|  | fresh fruit | vacuum freeze drying | hot air drying |
| --- | --- | --- | --- |
| D1 | 4.01±0.34 Aa | 3.63±0.27 Aa | 0.97±0.12 Bb |
| D2 | 20.08±2.34 Aa | 19.42±1.17 Aa | 11.62±0.54 Bb |
| D3 | 41.50±1.70 Aa | 38.32±3.91 Ab | 26.21±1.20 Bc |

chlorogenic acid content

|  | fresh fruit | vacuum freeze drying | hot air drying |
| --- | --- | --- | --- |
| D1 | 35.50±2.48 Aa | 15.62±0.68 Bb | 7.02±0.23 Cc |
| D2 | 43.72±1.51 Aa | 30.39±2.60 Bb | 19.74±0.51 Cc |
| D3 | 64.03±2.83 Aa | 55.67±1.61 Bb | 31.65±0.76 Cc |

flavone content

|  | fresh fruit | vacuum freeze drying | hot air drying |
| --- | --- | --- | --- |
| D1 | 12.56±1.04 Aa | 9.63±0.52 Bb | 8.80±0.88 Bb |
| D2 | 63.25±2.06 Aa | 58.71±1.44 Ab | 30.71±0.69 Bc |
| D3 | 113.11±6.31 Aa | 110.04±7.69 Aa | 42.44±4.12 Bb |

anthocyanin content

|  | fresh fruit | vacuum freeze drying | hot air drying |
| --- | --- | --- | --- |
| D1 | 1.18±0.17 Aa | 0.80±0.12 Aa | 0.00±0.00 Bb |
| D2 | 27.72±1.96 Aa | 25.19±0.38 Aa | 0.89±0.48 Bb |
| D3 | 32.51±0.89 Aa | 30.61±0.70 Aa | 4.56±1.00 Bb |

ABTS free radical scavenging ability

|  | fresh fruit | vacuum freeze drying | hot air drying |
| --- | --- | --- | --- |
| D1 | 21.03±0.12 Aa | 20.88±0.14 Aab | 20.71±0.19 Ab |
| D2 | 20.65±0.33 Aa | 20.69±0.10 Aa | 20.00±0.38 Ab |
| D3 | 20.81±0.20 Aa | 20.65±0.27 Aa | 19.15±0.35 Bb |

DPPH free radical scavenging ability

|  | fresh fruit | vacuum freeze drying | hot air drying |
| --- | --- | --- | --- |
| D1 | 25.25±1.44 Aa | 24.97±1.45 Aa | 18.56±3.12 Ab |
| D2 | 48.04±5.67 Aa | 27.59±0.74 Bb | 23.49±3.68 Bb |
| D3 | 29.60±3.47 Aa | 25.23±3.60 Aab | 22.39±2.37 Ab |

iron reducing force

|  | fresh fruit | vacuum freeze drying | hot air drying |
| --- | --- | --- | --- |
| D1 | 2.91±0.94 Aa | 2.35±0.68 Aa | 0.90±0.27 Bb |
| D2 | 41.84±2.54 Aa | 34.59±1.76 Bb | 17.50±1.84 Cc |
| D3 | 28.06±4.30 Aa | 24.41±2.59 Aab | 21.10±0.74 Ab |
