# Supplementary material for: Effect of vacuum freeze drying and hot air drying on dried mulberry fruit quality
Source: PLoS One. 2023 Jun 23;18(6):e0283303. doi: 10.1371/journal.pone.0283303 (PMC10289396; doi:10.1371/journal.pone.0283303)
Supplement: Attachment — Submitted filename: PONE-D-22-29075_R1reviewed .pdf [file pone.0283303.s003.pdf]

PLOS ONE

Effect of vacuum freeze drying and hot air drying on dried mulberry fruit quality

--Manuscript Draft--

**Manuscript Number:**	PONE-D-22-29075R1
**Article Type:**	Research Article
**Full Title:**	Effect of vacuum freeze drying and hot air drying on dried mulberry fruit quality
**Short Title:**	Different drying methods
**Corresponding Author:**	Hongjiao Li<br>NorthWest A&F University<br>Yangling, CHINA
**Keywords:**	Key words: Mulberry; Vacuum freeze drying; Hot air drying; Nutritional quality; The active ingredient
**Abstract:**	Two different drying methods (vacuum freeze-drying and hot-air drying) were used to dry mulberry of three varieties 'Baiyuwang'(D1), 'Longsang'(D2) and 'Zhongshen.1'(D3), and the fresh fruit of each variety was used as the control. The effects of different processing conditions on the physical characteristics, nutrients, functional components and antioxidant activity of mulberry fruit were analyzed. The results show that after different drying methods, after vacuum freeze-drying, the physical properties of dried mulberry fruit such as wettability, hygroscopic property and water retention, soluble protein, ascorbic acid and other nutrients, functional components such as polyphenols, resveratrol, chlorogenic acid and anthocyanin, and antioxidant activities such as DPPH free radical scavenging ability and ABTS free radical scavenging ability were superior to hot air drying ($P < 0.01$). It was concluded that vacuum freeze drying was more beneficial for retaining the original quality of mulberry than hot air drying. This study can provide a retaining theoretical basis for mulberry deep processing and comprehensive development and utilization.
**Order of Authors:**	li wang
	haichao wen
	ningwei yang
	hongjiao li
**Opposed Reviewers:**	
**Response to Reviewers:**	Reply to all comments point-by-point (Manuscript Number: PONE-D-22-29075)<br><br>The authors' replies are in blue.<br><br>COMMENTS FOR THE AUTHOR:<br>Reviewer 1#<br><br>1.The findings are not novel and previous studies have already demonstrated that vacuum freeze drying is a better method of drying, but the study has conducted in a very systematic way and has contribution to the mulberry fruit drying.<br><br>[Reply] Thank you very much for your comments. Yes, there have been many researches on fruit drying methods, but most of them focus on the influence of drying methods on fruit quality, or the advantages and disadvantages of different drying methods. This paper mainly studies the physical and chemical analysis, nutritional composition, functional composition and antioxidant capacity of fruits under different drying methods. This is one of the reasons for our research. We hope to use mulberry as the test material to get the difference of fruit quality under different drying methods.<br><br>2 The introduction of the manuscript is not adequately structured and need more information to be added.<br><br>[Reply] The introduction of this article has been added. Thank you for your valuable

Powered by Editorial Manager® and ProduXion Manager® from Aries Systems Corporation

comments.

3. The material methods are mentioned in detail, but there are some repetitions, which have compromised the quality of the methodology. In addition the authors should write materials and methods comprehensively.

[Reply] Thank you very much for your suggestion. We have rewritten the materials and methods.

4. The data is properly analyzed and have presented in well-illustrated figures, but results need more appropriate description and discussion, the authors should highlight the important findings.

[Reply] We have corrected the part of description and discussion, thank you for your suggestion.

5. In the abstract line 5 to 8 are weird, the authors need to rephrase the sentence.

[Reply] The abstract of this paper has been revised. please see line 12-19 of the "Revised Manuscript with Track Changes", Thank you for your valuable comments.

6. In Introduction line 4, the authors should paraphrase the sentence to give correct sense.

[Reply] Thank you very much for your suggestion. We have corrected it.

7. In Intro line 8 and 9 the sentence making no sense they should be rephrased.

[Reply] Thank you very much for your suggestion. We have corrected it.

8. The format of manuscript should be carefully rechecked to correct mistakes in the format.

[Reply] We have corrected it according to the manuscript format. Thank you for your comments.

9. English must go through a revision, there are some grammar mistakes that compromise the article.

[Reply] We have modified the English language appropriately. Thank you very much for your valuable suggestions. We will continue our efforts to improve our English in the future.

COMMENTS FOR THE AUTHOR:
Reviewer 2#

1.The paper is not written well scientifically (please see below!). The authors should maintain a standard English all through the paper taking care of the grammar.

[Reply] We have modified the English language appropriately. Thank you very much for your valuable suggestions. We will continue our efforts to improve our English in the future.

2. The authors should write a more proper introduction with proper literature review (information) and references.

[Reply] Thank you very much for your suggestion. The introduction part has been revised and appropriate references have been added. please see line 24-65 of the "Revised Manuscript with Track Changes"

3. The authors should mention the logic of using the given 3 cultivars of the mulberry fruit and should mention how they are different among themselves (it must be written in the introduction).

	[Reply] Thank you very much for your suggestions. We have revised the manuscript. In this paper, three mulberry varieties were planted in Dingzhou for 5 years. Bai Yu Wang was a white variety, Longsang was a northern black variety, and Zhong Shen 1 was a southern purple black variety. please see line 62-63 of the “Revised Manuscript with Track Changes”<br><br>4. Please write the references for all the information mentioned in the paper e,g ‘’Previous studies on hot air drying and vacuum freeze-drying of mulberry were carried out, but there was a lack of systematic study on the effects of hot air drying and vacuum freeze-drying on the comprehensive quality of different varieties of mulberry…..’’<br>Here the references are missing ??<br><br>[Reply] We have added the references for this part, Please see line 421-423 of the “Revised Manuscript with Track Changes”.<br><br>5. Please explain the ‘’Methods Section’’ properly and in detail. Vital information related to method is/are missing. E.g., What is the processing time taken of the fruits from the field to the actual processing of the samples (for the experiments)?<br><br>[Reply] Thank you very much for your suggestion. We have added this part. Please see line 68-69 of the “Revised Manuscript with Track Changes”.<br><br>6. Please write the full name of all the chemical used at the place of its first occurrence in the manuscript and later use the abbreviations e.g ABTS, DPPH, TPTZ. Authors have not mentioned the detail of the chemicals used.<br><br>[Reply] We have made some changes in the article, Please see line 71-73 of the “Revised Manuscript with Track Changes”. Thank you very much for your valuable advice.<br><br>7. Authors should clarify why they used different ‘’anti-oxidant assays’’ to measure the same thing? Please mention the logic and the benefit if any (also mention references)?<br><br>[Reply] References have been reviewed and cited, please refer to lines 489-490 of "Revised Manuscript with Track Changes".<br><br>8. In ‘’Materials and Method section’’ the authors have mentioned ‘’Mulberry (Baiyuwang (D1), Longsang (D2) and Zhongshen 1 (D3)) were picked from Dongsheng Ecological Park in Dingzhou City,….’’ The author should use the code D1, D2 and D3 in the abstract itself where they have mentioned the name of the different cultivar of the mulberry. After that the authors should stick to the code (no need to mention full name everywhere!).<br><br>[Reply] Thank you very much for your suggestions. We have used codes D1, D2 and D3 in the abstract, and will stick with them for the rest of the article.<br><br>9. In the manuscript, the authors have mainly written the results!! They must discuss their results (Discussion part) in proper and a better logical way based on their findings (results) and the known literature in this topic. The results and the discussion part must be well written and should make a better sense.<br><br>[Reply] Thank you very much for your suggestions. We have revised the manuscript. Please refer to the manuscript.<br><br>10.The ‘’Conclusion Section’’ should also be ‘’re-written’’ properly based on the results with future perspective etc.<br><br>[Reply] Thank you very much for your suggestion. We have corrected the content of the conclusion, please refer to the manuscript.<br><br><br>Once again, thank you very much for your comments and suggestions. Those comments are all valuable and very helpful for revising and improving our manuscript.

Additional Information:	
**Question**	**Response**
**Financial Disclosure**<br><br>Enter a financial disclosure statement that describes the sources of funding for the work included in this submission. Review the submission guidelines for detailed requirements. View published research articles from *PLOS ONE* for specific examples.<br><br>This statement is required for submission and **will appear in the published article** if the submission is accepted. Please make sure it is accurate.<br><br>**Unfunded studies**<br>Enter: *The author(s) received no specific funding for this work.*<br><br>**Funded studies**<br>Enter a statement with the following details:<br>• Initials of the authors who received each award<br>• Grant numbers awarded to each author<br>• The full name of each funder<br>• URL of each funder website<br>• Did the sponsors or funders play any role in the study design, data collection and analysis, decision to publish, or preparation of the manuscript?<br>• **NO** - Include this sentence at the end of your statement: *The funders had no role in study design, data collection and analysis, decision to publish, or preparation of the manuscript.*<br>• **YES** - Specify the role(s) played.<br><br>* typeset	Hongjiao Li ,Natural Science Foundation of Hebei Province (C2019204257)<br>Youth Fund for Science and Technology Research Projects of Colleges and Universities in Hebei Province (QN2019052)<br>Funders play a certain role in research design, data collection and analysis, publication decision or manuscript preparation.
**Competing Interests**<br><br>Use the instructions below to enter a competing interest statement for this submission. On behalf of all authors, disclose any competing interests that could be perceived to bias this work—acknowledging all financial support and any other relevant financial or non-financial competing interests.	There is no competition in this article.

This statement is required for submission and **will appear in the published article** if the submission is accepted. Please make sure it is accurate and that any funding sources listed in your Funding Information later in the submission form are also declared in your Financial Disclosure statement.<br><br>View published research articles from *PLOS ONE* for specific examples.<br><br>**NO authors have competing interests**<br><br>Enter: *The authors have declared that no competing interests exist.*<br><br>**Authors with competing interests**<br><br>Enter competing interest details beginning with this statement:<br><br>*I have read the journal's policy and the authors of this manuscript have the following competing interests: [insert competing interests here]*<br><br>* typeset	
**Ethics Statement**<br><br>Enter an ethics statement for this submission. This statement is required if the study involved:<br><br>• Human participants<br>• Human specimens or tissue<br>• Vertebrate animals or cephalopods<br>• Vertebrate embryos or tissues<br>• Field research<br><br>Write "N/A" if the submission does not require an ethics statement.<br><br>General guidance is provided below. Consult the submission guidelines for detailed instructions. **Make sure that all information entered here is included in the**	No

**Methods section of the manuscript.**<br><br>**Format for specific study types**<br><br>**Human Subject Research (involving human participants and/or tissue)**<br>• Give the name of the institutional review board or ethics committee that approved the study<br>• Include the approval number and/or a statement indicating approval of this research<br>• Indicate the form of consent obtained (written/oral) or the reason that consent was not obtained (e.g. the data were analyzed anonymously)<br><br>**Animal Research (involving vertebrate animals, embryos or tissues)**<br>• Provide the name of the Institutional Animal Care and Use Committee (IACUC) or other relevant ethics board that reviewed the study protocol, and indicate whether they approved this research or granted a formal waiver of ethical approval<br>• Include an approval number if one was obtained<br>• If the study involved *non-human primates*, add *additional details* about animal welfare and steps taken to ameliorate suffering<br>• If anesthesia, euthanasia, or any kind of animal sacrifice is part of the study, include briefly which substances and/or methods were applied<br><br>**Field Research**<br><br>Include the following details if this study involves the collection of plant, animal, or other materials from a natural setting:<br>• Field permit number<br>• Name of the institution or relevant body that granted permission	
**Data Availability**<br><br>Authors are required to make all data underlying the findings described fully available, without restriction, and from the time of publication. PLOS allows rare exceptions to address legal and ethical concerns. See the PLOS Data Policy and FAQ for detailed information.	No - some restrictions will apply

A Data Availability Statement describing where the data can be found is required at submission. Your answers to this question constitute the Data Availability Statement and **will be published in the article**, if accepted.<br><br>**Important:** Stating 'data available on request from the author' is not sufficient. If your data are only available upon request, select 'No' for the first question and explain your exceptional situation in the text box.<br><br>Do the authors confirm that all data underlying the findings described in their manuscript are fully available without restriction?	
**Describe where the data may be found in full sentences. If you are copying our sample text, replace any instances of XXX with the appropriate details.**<br><br>• If the data are **held or will be held in a public repository**, include URLs, accession numbers or DOIs. If this information will only be available after acceptance, indicate this by ticking the box below. For example: *All XXX files are available from the XXX database (accession number(s) XXX, XXX.*).<br>• If the data are all contained **within the manuscript and/or Supporting Information files**, enter the following: *All relevant data are within the manuscript and its Supporting Information files.*<br>• If neither of these applies but you are able to provide **details of access elsewhere**, with or without limitations, please do so. For example:<br><br>*Data cannot be shared publicly because of [XXX]. Data are available from the XXX Institutional Data Access / Ethics Committee (contact via XXX) for researchers who meet the criteria for access to confidential data.*<br><br>*The data underlying the results*	All relevant data are within the manuscript and its Supporting Information files

presented in the study are available from (include the name of the third party and contact information or URL).<br>• This text is appropriate if the data are owned by a third party and authors do not have permission to share the data.<br><br>* typeset	
Additional data availability information:	Tick here if the URLs/accession numbers/DOIs will be available only after acceptance of the manuscript for publication so that we can ensure their inclusion before publication.

Powered by Editorial Manager® and ProduXion Manager® from Aries Systems Corporation

Dear Editor,

I submit a manuscript entitled "Effects of Vacuum Cold Air Drying and Hot Air Drying on mulberry Fruit Quality" for your consideration for publication as a research paper in a special issue of Plos One.

The manuscript is intended for exclusive publication in this journal. There is no conflict of interest in submitting this manuscript, which was approved for publication by all authors. On behalf of my coauthor, I would like to state that the work described is original research that has not previously been published and is not under consideration for publication elsewhere, in whole or in part. All the authors listed approved the attached manuscript.

Morusalbal. belongs to Moraceae, and its fruit is mulberry. The fruit of mulberry contains more nutrients, such as organic acids, sugars, antioxidant substances, etc., so it has high edible value. However, mulberry is a berry, and its fruit is highly perishable, which is not conducive to postharral storage, long-distance transportation and sales.In view of the storage intolerance of mulberry fruit, its fruits are often processed into different products.

The development trend of modern food processing technology is one of the most important is to maintain food nutrition and flavor. Common mulberry wine, mulberry products are dried fruit, mulberry fruit drinks, etc., and dried mulberry is one of the main processing, forms that can greatly solve the problem of mulberry not being resistant to storage, the choice of drying technology and equipment for drying has a great impact on the nutritional quality of the product.

Mulberry is the ripe fruit of mulberry tree, black, white and other colors, and fresh food to purple-black is used as a tonic. Mulberry is a healthy fruit with the same homology of medicine and food listed by the Ministry of Health. Mulberry contains trace elements such as iron, calcium, zinc and manganese required by the human body, as well as pharmacological components such as polysaccharides, amino, acids anthocyanin, resveratrol and flavones. It is regarded as a good health care product in both traditional and modern medicine. Due to the special growing environment of mulberry, mulberry

fruit has the characteristics of natural growth and no pollution, so mulberry is also called "folk holy fruit".

The vacuum freeze drying and hot air drying on the influence of the mulberry fruit "is to use two different drying methods (vacuum freeze drying and hot air drying) on" Bai Yuwang ", "dragon Shen in mulberry 'and' 1 'three varieties of mulberry to dry, analysis of different drying process conditions on the physical properties of the mulberry fruit, nutrient and functional components and the effect of antioxidant activity,Selecting the best drying method can keep the quality of fruit to the maximum extent, and provide a certain theoretical basis for the deep processing and comprehensive development and utilization of mulberry.

We hope that this will serve as a good contribution to a regular document on horticulture. Thank you for your consideration, and I look forward to your reply.

Sincerely,

Hongjiao Li
A lecturer
College of Forestry, Agricultural University of Hebei, Hebei Key Laboratory of Forest Germplasm Resources and Forest Protection, Baoding 071000, China
Email：lihongjiao0103@126.com
Phone：86+15128968966

Manuscript Click here to access/download;Manuscript;Manuscript.docx

Effect of vacuum freeze drying and hot air drying on dried mulberry fruit quality

Li Wang[1,2], Haichao Wen[1,2], Ningwei Yang[1,2], Hongjiao Li[1,2*]

1 College of Forestry, Agricultural University of Hebei

2 Key Laboratory of Germplasm Resources of Forest Tree and Forest Protection of Hebei Province, Baoding 071000, China

* lihongjiao0103@163.com

Abstract

Two different drying methods (vacuum freeze-drying and hot-air drying) were used to dry mulberry of three varieties 'Baiyuwang'(D1), 'Longsang'(D2) and 'Zhongshen.1'(D3), and the fresh fruit of each variety was used as the control. The effects of different processing conditions on the physical characteristics, nutrients, functional components and antioxidant activity of mulberry fruit were analyzed. The results show that after different drying methods, after vacuum freeze-drying, the physical properties of dried mulberry fruit such as wettability, hygroscopic property and water retention, soluble protein, ascorbic acid and other nutrients, functional components such as polyphenols, resveratrol, chlorogenic acid and anthocyanin, and antioxidant activities such as DPPH free radical scavenging ability and ABTS free radical scavenging ability were superior to hot air drying ($P < 0.01$). It was concluded that vacuum freeze drying was more beneficial for retaining the original quality of mulberry than hot air drying. This study can provide a retaining theoretical basis for mulberry deep processing and comprehensive development and utilization.

Key words: Mulberry; Vacuum freeze drying; Hot air drying; Nutritional quality; The active ingredient

1 Introduction

Mulberry, also known as mulberry fruit, mulberry jujube, black mulberry, etc., is the mature ear of mulberry (Morus alba L.). Mulberry has high nutritional value and is rich in amino acids [1], proteins [2], vitamins [3], minerals and other nutritional elements. It is also rich in polysaccharides [4-5], alkaloids [6], flavonoids, resveratrol [7], anthocyanins [8] and other functional active ingredients, which have the medicinal value of enhancing immunity, antioxidants, regulating blood lipids and lowering blood sugar [9-11]. It is known as "the third generation of new fruit", and by the Ministry of Health as a drug and food dual-use plant resource [12].

Mulberry is a berry, that is tender and juicy, sweet and sour, and most mature in late spring and early summer with high temperature and high humidity. Due to the high water content of its fruits, it brings great difficulties in storage, transportation and preservation. In addition, it is easy to damage and rot after harvest, difficult to store, and its shelf life is short, which greatly limits the development of the mulberry industry. Therefore, dry processing of mulberry fruit is an important way to reduce fresh fruit loss and increase product value. There are relatively many drying methods available, but drying and direct drying are usually used for mulberry fruit drying at present, both of which have advantages and disadvantages. Therefore, modern high-tech food processing technology is applied to dry mulberry, product production to extend the shelf life of food [13]. It is of great significance to improve the processing level of dried mulberry products and promote the industrialization of mulberry.

An important development trend of modern food processing technology is to maintain the nutrition, color, and flavor of food to the maximum extent. Common mulberry products include mulberry wine, dried mulberry fruit, mulberry drinks, etc. Dry processing is one of the main processing forms of mulberry, which can greatly solve the problem of intolerant storage of mulberry, and the selection of drying technology and equipment has a great influence on the nutritional quality of dried products. Hot air drying is the most commonly used drying method, but the drying speed is slower and the drying time is longer [14]. Vacuum freeze-drying is a process of removing water or other solvents from frozen biological products through sublimation. It is a raw material drying method for obtaining high quality biological products, which has the characteristics of maintaining the original biological activity, color and shape, fast sample dissolution rate and low residual moisture [15].

A large number of experimental studies have been conducted on the drying characteristics of mulberry under various drying methods at home and abroad. Mahmood *et al*. [16] used natural drying and sun drying treatments for different varieties of mulberry, and the results showed that drying conditions had significant effects on the polyphenol and antioxidant properties of mulberry. The research results of Ester *et al*. [17] showed that freeze-drying treatment made samples have better

texture and color. Previous studies on hot air drying and vacuum freeze-drying of mulberry have been conducted, but there is a lack of systematic studies on the effects of hot air drying and vacuum freeze-drying on the comprehensive quality of different varieties of mulberry [18]. In this study, hot air drying and vacuum freeze-drying techniques were used to explore the effects of drying methods on the physical properties, nutritional quality, functional components and antioxidant activity of dried D1, D2 and D3 of the three mulberry varieties, which were all planted in Dingzhou for 5 years, and D1 was a white variety. D2 is a northern black variety and D3 is a southern purple black variety. To provide a theoretical reference for choosing different mulberry drying treatment methods, the changes in dried fruit quality were discussed.

2. Materials and Methods

Mulberries (D1, D2 and D3) were all picked in Dongsheng Ecological Park, Dingzhou City, Hebei Province. Mulberry was picked manually and each sample was put into a separate bag after picking. The mulberry was tested immediately after being transported to the laboratory.

Vacuum freeze-drying and hot air drying are adopted.

The chemical reagents used in the whole study were of analytical grade. 2, 2'-Azino-bis(3-ethylbenzothiazoline-6-sulfonic acid), 2,2-diphenyl-1-picrylhydrazyl (EPR spectroscopy), 2,4,6-tris(2-pyridyl)-s-triazine and Folin-Ciocalteu were purchased from Solarbio, and bovine serum protein, glycine, rutin, resveratrol and chlorogenic acid standards were purchased from Sourleaf Biological Co., Ltd. Reagents such as anthrone, concentrated sulfuric acid, ninhydrin, sodium hydroxide, ethanol, sodium carbonate and sucrose were purchased from Sinopharm Chemical Reagent Co., Ltd.

2.1 Drying Process

The fresh mulberry was dried in a vacuum freeze dryer and air drying oven. Two different drying methods were used to take a fixed weight sample (150 g). All fresh mulberries were dried until they reached a constant weight. The following drying modes were used for sample preparation. All fresh mulberry fruits were dried until they reached a constant weight. The following drying mode was used for the preparation of samples.

2.1.1 Vacuum freeze-drying

Fresh mulberry (150 g) was completely frozen in a deep refrigerator at -50°C (24 hours) and then dried. Then, a vacuum freeze-dryer (FDU-1200) was used to dry for 48 h in a vacuum freeze-dryer under 20 pA at a constant temperature of -50°C.

2.1.2 Hot air drying

Fresh mulberry was spread on a perforated steel plate and dried in hot air at 60℃ for 48 h.

2.2 Physical and chemical analysis

Wettability was measured by referring to the method of Jumah *et al*. [18]. Then, 100 mL distilled water was added to a 250 mL beaker, 0.5 g mulberry powder was evenly spread on the water surface, and the time (s) required for all the powder to be wetted by water was recorded immediately. The test was repeated three times, and the average value was taken to represent the wettability of dried mulberry powder.

The moisture absorption method of a reference person such as Zhao [19] takes mulberry 3 g dried fruit powder, evenly spread in clean in a petri dish, and then puts the glass dish in an enamel disc, adding enough and saturated sodium chloride solution, after the two layers of sealing surface with plastic wrap and place for 7 days, accurate said in petri dishes and according to the formula to calculate the quality with the sample moisture absorption. The formula is as follows:

$$\text{Hygroscopicity (\%)} = \frac{\frac{m}{M+m}\times 100}{1+m/M}$$

where m is the weight change of mulberry powder before and after (g).

M: initial mass of mulberry powder (g).

The method of Hameed *et al.* [20] was used for determination by hydraulic reference. Mulberry leaf powder (0.5 g) was accurately weighed into a 10 mL centrifuge tube, 10 mL distilled water was added, thoroughly mixed for 10 min, and then centrifuged at 5000 R/min for 10 min. The supernatant was discarded, the residual water on the wall of the centrifuge tube was dried with filter paper, and

the mass was weighed. This was repeated three times, and the average value and standard deviation were used for drawing. The formula is as follows:

Hydraulic（g/g) = (M-m)/m

where M is the mass of dried fruit powder after centrifugation (g).

m: Initial mass of dried fruit powder (g).

The bulk density was measured according to the method of Sowbhagya *et al*. [21]. A certain amount of mulberry powder was put into a 10 mL centrifuge tube and shocked until the mulberry powder was just filled to the calibration line of the volumetric bottle. The bulk density (DO) of the powder was calculated by the following formula:

do (g/m L) = (M-m)/10

where M is the total weight of the fruit powder and centrifuge tube (g).

m: Test tube weight (g).

According to the method of Hameed *et al*. [22], 0.2 g of mulberry powder was placed in a centrifuge tube, 10 mL of distilled water was added, and the mixture was stirred evenly. After centrifugation at 5000 R/min for 5 min, 5 mL of the obtained supernatant was placed into a glass Petri dish, which was placed in an oven, and dried at 105°C for 3 h, and the total mass after drying was weighed. The formula is as follows:

solubility (%) = $(M-m) \times 2 \times 100/m_1$

where M is the total weight of the dried Petri dish and dissolved fruit powder (g).

m: Net weight of Petri dish (g).

m_1: Initial weight of mulberry powder (g).

2.3 Nutrient content

Soluble protein was determined according to the method of Bradford [23]. The mulberry sample (0.1 g) was accurately weighed and placed in a mortar with 5.0 mL H_3PO_4 buffer (pH=7.8), and fully ground. Then the mortar was rinsed with 5.0 mL of the above buffer and poured into a 10.0 mL centrifuge tube. The samples were centrifuged at a constant temperature of 10000 RMPs for 20 min at 4°C. Then, 1.0 mL of the supernatant was aspirated, 5.0 mL of Coomassie brilliant blue G-250 solution was added, and the absorbance value was measured at 595 nm after 2 min of mixing.

The soluble sugar content was determined according to the method of Candida [24].The mulberry sample of 0.1 g was accurately weighed and ground thoroughly with 10.0 mL distilled water in a mortar, placed in a 10 mL centrifuge tube, sealed and heated in a boiling water bath for 30 min.The heated solution was filtered through gauze, the rinsed residue was filtered repeatedly with distilled water, and the volume was stabilized into a 25 mL volumetric flask. Then, 1.5 mL of distilled water,

0.5 mL of anthrone, ethyl acetate reagent and 5.0 mL of concentrated sulfuric acid were added to the test tube in sequence and mixed with shock. The absorbance value was measured at 630 nm wavelength after the sample was cooled to room temperature. Finally, the soluble sugar content of the samples was calculated according to the standard curve and formula.

The content of free amino acids was determined by reference to the ninhydrin chromogenic method [25]. First, the mulberry sample was weighed by an analytical balance and ground into a slurry in a mortar. Then, 5.0 mL distilled water was added to the mortar and placed into the centrifuge tube. Then 5.0 mL distilled water was added to rinse the mortar and poured into the centrifuge tube. Ultrasonic extraction was carried out at 40°C for 20 min using an ultrasonic cleaning machine. After extraction, it was centrifuged at 5000 rpm for 25 min, and 1.0 mL of the supernatant was absorbed by a pipetting nozzle, Then, 2.0 mL of H_3PO_4 buffer with pH 6.0 was added, and 3.0 mL of ninhydrin solution was added after full shaking and standing for 5 min. The centrifuge tube was sealed and heated in a boiling water bath for 30 min. After cooling, the absorbance was measured at 560 nm, and the amino acid content was calculated according to the regression equation.

Determination of ascorbic acid (VC) content: The method of Habib *et al.* [26] was used for determination.

2.4 Content of functional components

The content of flavonoids in mulberry was determined by the $AlCl_3$ chromogenic method [27]. First, a 0.2 g mulberry sample was accurately weighed by an analytical balance, and 5.0 mL 70% C_2H_5OH was added according to a material to liquid ratio of 1:25, and placed into a centrifuge tube. Then, an ultrasonic cleaning machine was used for extraction for 40 min at a constant temperature of 25 ℃. The extracted extract was centrifuged at 7000 RPM for 10 min. For determination, 1.0 mL of supernatant was taken, 0.4 mL of 50% $NaNO_3$ solution and 0.4 mL of 10% $AlCl_3$ solution were added, the mixture was thoroughly shaken and left for 5 min, and 4 mL of 4% NaOH solution was added. A constant volume of 70% C_2H_5OH was used to 10.0 mL. After standing for 10 min at room temperature, the absorbance value was measured at a wavelength of 415 nm. Finally, the content of flavonoids in the samples was calculated according to the standard curve.

The content of polyphenols was determined by the Folin-Ciocalteu colorimetric method [28]. First, a 0.1 g mulberry sample was accurately weighed by an analytical balance, and 10.0 ml 50% C_2H_5OH was added according to a solid-liquid ratio of 1:100. The samples were extracted by ultrasonication for 40 min at a constant temperature of 25°C. This was followed by centrifugation at 7000 rpm for 10 min. After centrifugation, 1.0 mL of the supernatant was taken, 1.0 mL of folinol reagent was added, thoroughly shaken and mixed, and the mixture was allowed to stand for 5 min at

room temperature. Then, 4.0 ml 20% $NACO_3$ solution was added and heated in a 50°C water bath for 1 hour. After cooling to room temperature, the absorbance value was measured at 760 nm wavelength. Finally, the content of polyphenols in the samples was calculated according to the standard curve regression equation.

The resveratrol content was determined by the C_2H_5OH extraction method [29]. A mulberry sample of 0.2 g was accurately weighed, and 60% C_2H_5OH (3.0 mL) was added according to a solid-liquid ratio of 1:15, and placed into a 10.0 mL centrifuge tube. After shaking well, the mulberry sample was heated in a water bath at 60°C for 1 h, and the mulberry sample was kept away from light during the whole process. After filtration, samples were centrifuged at 7000 rpm for 10 min. For determination, 0.5 mL of supernatant was diluted 10 times with the above concentration of C_2H_5OH, the absorbance value was measured at 305 nm wavelength, and the content of resveratrol was calculated according to the standard curve and regression equation.

The chlorogenic acid content was determined by the Fe2+ chromogenic method [30].The 0.2 g sample was accurately weighed, and 10.0 ml 60% CH_3OH was added as the extraction agent according to the solid-liquid ratio of 1:5. The samples were extracted by ultrasound for 30 min.After simple filtration, 60% CH_3OH was used to a constant volume of 15.0 mL, 5.0 mL of constant volume extract was taken, 0.5 mL of 0.2 mol/L $FeCl_3$ solution was added, and the mixture was shaken and left for 60 min.The absorbance value was measured at a wavelength of 755 nm, and the chlorogenic acid content was calculated according to the standard curve and regression equation.

The anthocyanin content in mulberry was determined by the pH difference method [31]. A 0.2 g mulberry sample was accurately weighed by an analytical balance, 4.0 ml of 60% acidified C_2H_5OH was added according to a 1:20 solid-liquid ratio, and the whole process was kept away from light. Then the samples were placed in a 60°C water bath and heated for 2 h. The samples were allowed to cool to room temperature. Then 1.0 mL supernatant and 9.0 mL H_3PO_4 buffer with pH=1.0 and pH=4.5 were added to two 25 mL vials, shaken well, and left in the dark for 2 hours. The absorbance values were measured at wavelengths of 520 nm and 700 nm.

2.5 Antioxidant properties

DPPH free radical scavenging ability, ABTS free radical scavenging ability and iron reducing power of mulberry [32].

The DPPH radical scavenging ability was determined by the DPPH radical scavenging method. A mulberry sample of 0.1 g was accurately weighed by an analytical balance, and 50% C_2H_5OH (0.5 mL) was added according to a solid-liquid ratio of 1:50. After ultrasonic extraction, the volume was fixed to 10.0 mL for later use. For measurement, 0.5 mL of the extract was taken, 2.0 mL of DPPH

reaction solution was added, and the reaction was shaken and kept away from light for 30 min. The light absorption value was measured at a wavelength of 517 nm. In the control group, the same volume of CH_3OH was used instead of the extract, and the final results were expressed as μmol/LTrolox equivalent antioxidant capacity. The measured absorbance value was taken as the abscissa, and the concentration of Trolox standard solution was taken as the ordinate. The standard curve was drawn, and the regression equation was calculated.

In the determination of ABTS free radical scavenging ability, the method of preparing the extract was the same as that of DPPH, and ABTS+ working solution was prepared in advance:10.0 ml ABTS solution of 7 mmol/L and 5.0 potassium persulfate solution of 7.35 mmol/L were mixed, placed in the dark for 16 h at room temperature, and then prepared for use. The absorbance was diluted to 0.70 ± 0.02 at 734 nm when used. At the time of measurement, 0.5 mL of the extract was taken, 2.0 mL ABTS+ working solution was added, and the reaction was kept away from light for 6 min after shaking. The absorbance value was measured at a wavelength of 734 nm. The final results were expressed as μmol/LTrolox equivalent antioxidant capacity. The measured absorbance value is the abscissa, the concentration of Trolox standard solution is the ordinate, the standard curve is drawn, and the regression equation is calculated.

Iron reducing capacity was determined by the FRAP method. The method of preparing the extract in the determination is the same as that in DPPH. Before the test, the TPTZ working solution was prepared in advance: CH_3COONa solution pH=3.6. Then, 300 mmol/L, 10 mmol/L, TPTZ solution, and 20 mmol/L, $FeCL_3 \cdot 6H_2O$ solution were mixed according to a volume ratio of 1:1:1. For measurement, 0.5 mL of extract was taken, 1.0 mL of distilled water and 1.8 mL of TPTZ working solution were added, and the reaction was kept away from light for 10 min. The absorbance value was measured at a wavelength of 593 nm, and the final result was expressed as the antioxidant capacity of μmol/L Trolox. The measured absorbance value is the abscissa, and the concentration of Trolox standard solution is the ordinate. The standard curve was drawn and the regression equation was calculated.

2.6 Statistical Analysis

A completely randomized design (CRD) was used to analyze the physicochemical properties, nutrients, functional components and antioxidant properties of mulberry fruit. One-way analysis of variance (P <0.05) was used to analyze significant differences between treatments. The parameters of chemical and antioxidant properties of different varieties of mulberry were repeated three times in these studies.

3 Results and discussion

Table 1 shows the physical properties of mulberry fruit under different drying modes (vacuum freeze-drying and hot-air drying). Figure 1 shows the changes in the nutrient composition of mulberry fruit under different drying modes. Figure 2 shows the changes in the functional components of mulberry fruit under different drying modes. Figure 3 shows the variation in the antioxidant capacity of mulberry fruit under different drying modes.

3.1 Changes in physical characteristics under different drying treatments

Table 1 describes the effects of the different drying processes used in this study on fruit wettability, hygroscopicity, water retention, bulk density and solubility. In terms of physical and chemical properties, fruit wettability, moisture absorption, water retention, bulk density and solubility all reflect the effects of drying processes on fruit quality. The results in Table 1 show that the physicochemical properties of different mulberry varieties after vacuum freeze-drying were better than those after hot air drying, which indicated that the use of exhaust heat from the condenser as the heat source could significantly accelerate the rate of water content decline. These data indicate that an important feature of vacuum freeze dryers is the efficient use of energy to reduce moisture content compared to other drying methods.

Moisture content is a very important characteristic of freeze-dried foods, as it affects the appearance, texture and taste of the product. Moisture content also affects the freshness and shelf life of products. A high moisture content will make it easy for bacteria, mold and yeast to multiply, and the food will change. The vacuum freeze-drying process removes moisture from the sample. In terms of the physical properties of mulberry, the fruit powder with packing density, hygroscopic property, wettability and solubility after vacuum freezing is better than that after hot air drying, which is related to the hardening phenomenon on the surface of the mulberry powder after hot air drying and the inability to form a large number of micropores inside the fruit powder, which is consistent with the results of Caparino [33].

Table 1

Effects of different drying methods on the physical properties of mulberry fruit powder

Varieties	process mode	Wettability(s)	Hygroscopicity(%)	water-holding power(g/g)	bulk density(g/mL)	Solubleness(%)
D1	vacuum freeze drying	241.00±22.07aA	9.13±1.59aA	2.09±0.05aA	0.46±0.07aA	28.15±14.68aA
	hot air drying	55.00±8.50bB	4.69±2.90bB	1.69±0.33aA	0.53±0.14bB	35.03±0.84aA
D2	vacuum freeze drying	65.00±7.23aA	10.41±0.47aA	2.09±0.09aA	0.54±0.09aA	38.41±2.50aA

	hot air drying	27.00±5.86bB	7.74±0.72bB	1.38±0.02bB	0.52±0.07aA	41.57±2.41aA
D3	vacuum freeze drying	144.00±20.60aA	10.35±0.68aA	2.17±0.01aA	0.47±0.21aA	37.75±0.81aA
	hot air drying	110.00±10.50aA	7.39±0.82bB	1.24±0.00bB	0.66±0.59bB	43.48±9.74aA

Note: Lowercase letters indicate the significance of differences between different treatments of the same variety (*P*<0.05), uppercase letters indicate the significance of differences between different treatments of the same variety (*P*<0.01).

3.2 Changes in nutrient content under different drying treatments

The results of Figure 1 show that after vacuum freeze-drying and hot-air drying, the nutrient content of mulberry fruits of the three varieties changed. Compared with fresh mulberry fruit, the content of mulberry fruit showed a decreasing trend after different drying treatments, but the retention of these components by vacuum freeze-drying was better than that by hot air drying, which may be because hot air drying would promote the Maillard reaction and Strecker degradation reaction of amino acids in mulberry fruit, resulting in the loss of amino acids [34].The reason why vacuum freeze-drying reduces the content of amino acids may be because freezing can prevent proteins from degrading into amino acids [35]. The research results of Paramanandam *et al*. [36] found that the content of amino acids and proteins in fruits under hot air drying was higher, which is different from the research results in this paper and needs to be further verified. In addition, the research results of Harguindeguy *et al*. [37] show that different drying processes have an impact on the ascorbic acid content, phenolic compounds and antioxidant properties of food.

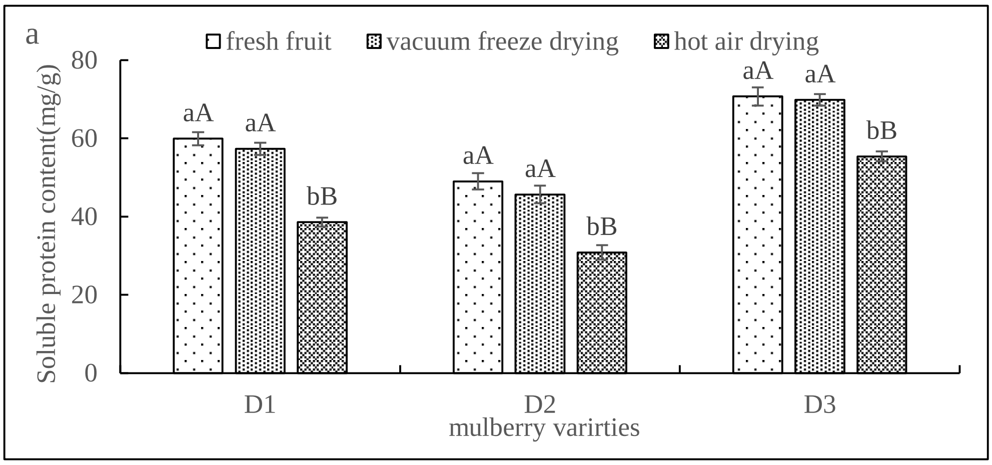

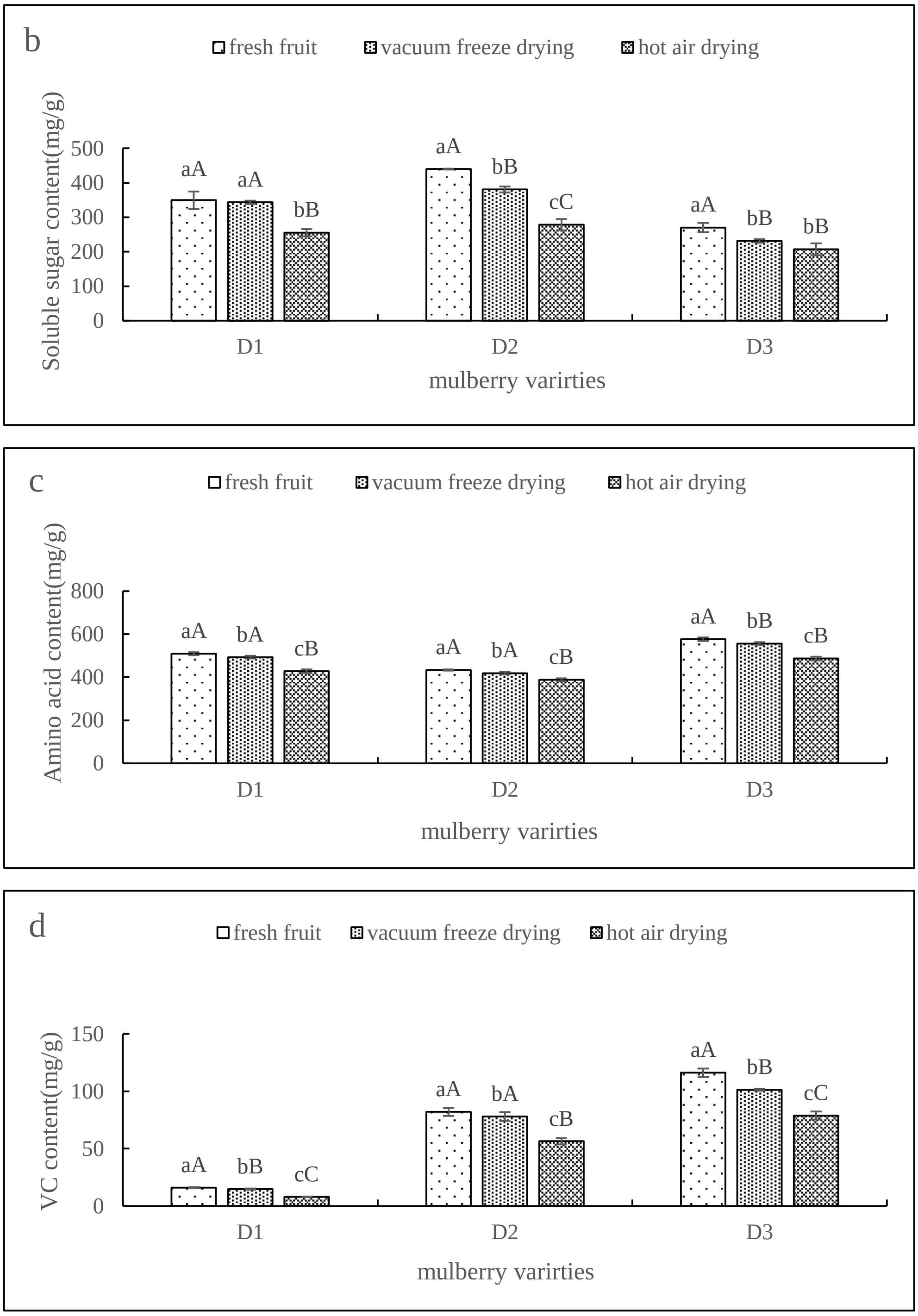

Fig.1 (a, b, c, d) shows the changes in soluble protein, soluble sugar, amino acid and ascorbic acid contents of fruits under different drying treatments (error bars indicate the standard error of the average). Different letters a-c in the same column indicate significant differences ($P < 0.05$).

3.3 Content changes of functional components under different drying treatments

Figure. 2 shows the changes in the functional components of the three mulberry fruits under different drying modes. According to the data, compared with fresh mulberry fruit, the contents of total phenols, total flavonoids, ascorbic acid and neo-chlorogenic acid under vacuum freeze-drying

were significantly higher than those under hot air drying after different drying treatments. This was because the functional components such as polyphenols, flavonoids and vitamins in mulberry were affected by light, temperature, oxygen partial pressure, water activity and other factors during the drying process, and oxidation, aggregation or decomposition occurred, resulting in changes in the content of functional components [38-40]. In addition, under the conditions of low temperature and low oxygen partial pressure, the activity of oxidase is low. Therefore, thermally sensitive components such as polyphenols, flavonoids and ascorbic acid easily undergo enzymatic oxidation and have better retention under this dry condition. Hot air drying destroys heat sensitive components, such as flavonoids [41-42]. The research results of Angela *et al*. [43] showed that compared with hot-air drying, the content of flavonoids and phenolic acids was higher under freeze-drying. Tan *et al*. [44] found that the content of anthocyanins in fresh fruits was the highest and decreased with increasing drying temperature. According to the analysis, cyanidin-3-O-glucoside and delphinidin-3-O-galactoside were the main anthocyanins in flesh-and-blood peaches, but during the whole drying process, the content of delphinidin-3-O-galactoside decreased continuously, which was consistent with the results of this study.

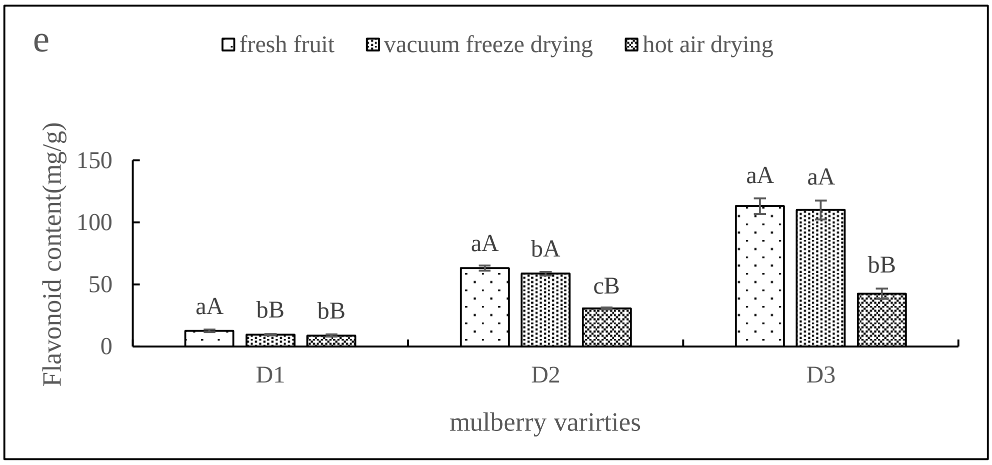

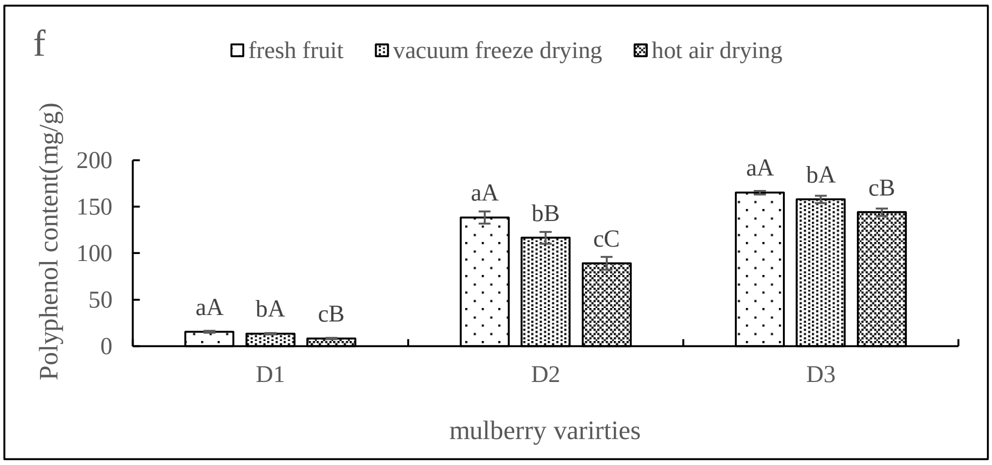

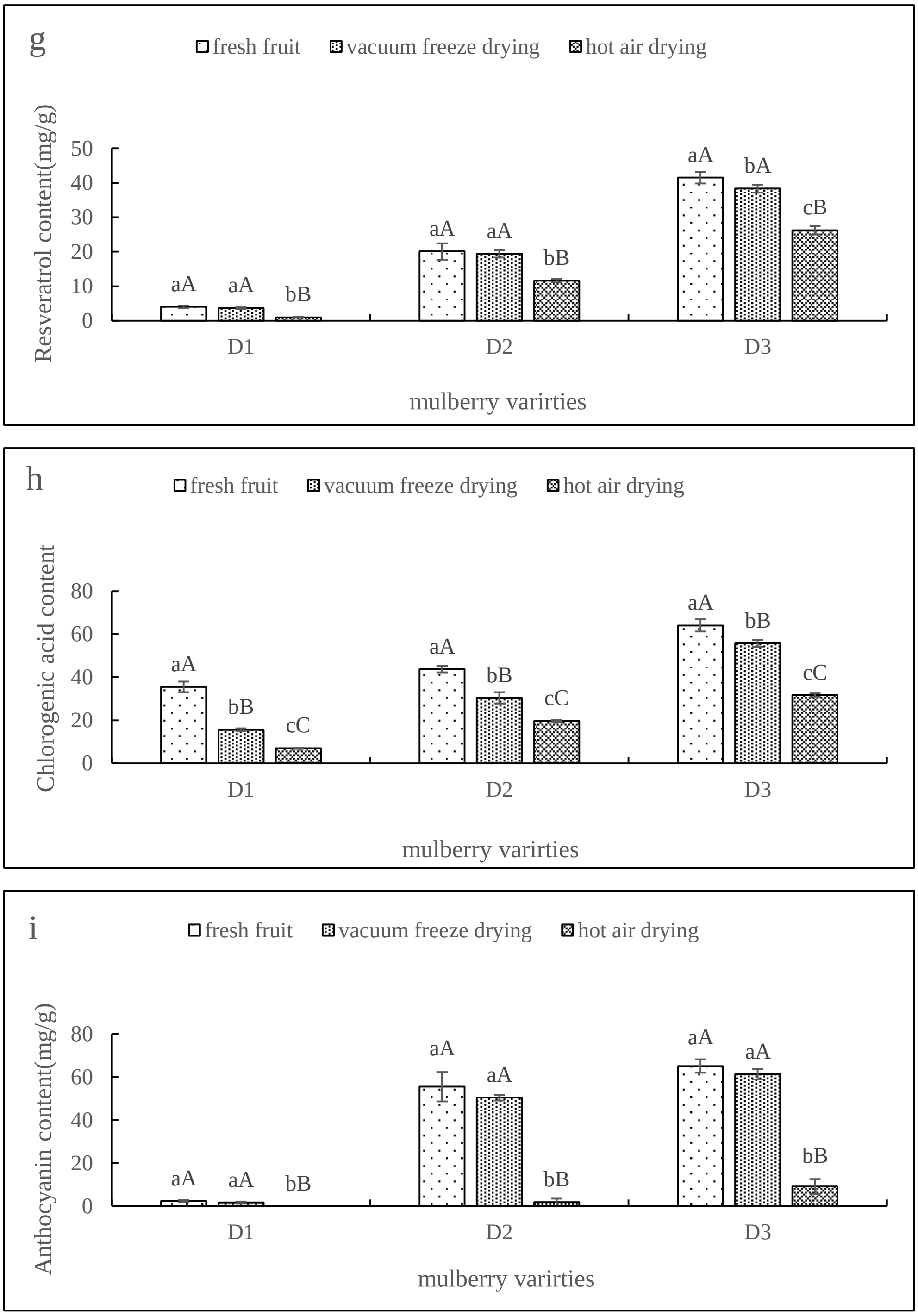

Fig 2 (e, f, g, h) shows changes in the contents of flavonoids, polyphenols, resveratrol, chlorogenic acid and anthocyanins in fruits under different drying treatments (error bars indicate the standard error of the average). Different letters a-c in the same column indicate significant differences ($P < 0.05$).

3.4 Changes in antioxidant properties under different drying treatments

Total phenols and flavonoids were the main functional components of mulberry, and phenolic compounds composed of chlorogenic acid and its derivatives were dominant. Polyphenol compounds contain multiple phenolic hydroxyl groups and have strong reducing properties. Their structural

properties are the main reason for the antioxidant activity of these compounds. They achieve antioxidant activities mainly by directly scavenging free radicals or interacting with metal ions. The results showed that the antioxidant activity of mulberry under vacuum freeze-drying was stronger, which might be due to the better retention and reducibility of phenolic compounds under vacuum freeze-drying. Ozkan *et al*. [45] reported a similar trend in their findings. This was also confirmed by the results of Zhang *et al*. [46], who found that the scavenging activity of chlorogenic acid on DPPH and ABTS free radicals reached 92.4% and 99.4% respectively. Chen *et al*. [47] determined the total phenols, flavonoids, vitamin C and antioxidant capacity of 25 mulberry samples from 6 provinces, and the results showed that D3 had the highest health care value and the highest active compound.content The data in the same figure show that the antioxidant activity of mulberry under vacuum freeze-drying is high, while that under hot-air drying is low. Therefore, the contents of total phenols, flavonoids and antioxidant substances in mulberry have great influence on the antioxidant capacity of mulberry, and the contents of functional components in mulberry fruits, especially phenolic substances, are affected by different drying methods. The drying method also determines its oxidation resistance.

In conclusion, both hot air drying and vacuum freeze-drying had significant effects on the dry quality characteristics of mulberry fruit, and vacuum freeze-drying could more effectively retain the nutritional quality, functional components and antioxidant activity of fresh mulberry fruit. This study provides an effective reference for the further development and utilization of mulberry products.It can also provide some reference for further study on the effect of drying methods on mulberry fruit drying.Abbaspour, Igor and Duan *et al*. [47-49] stated that freeze-drying has the lowest color and shrinkage rate of fruits, the highest content of bioactive compounds, and better rehydration performance, and can maintain the original quality to the maximum extent, so it has broader application prospects in food, medicine, biological products and other fields. However, the relationship between the drying method and fruit characteristics should be considered at the same time. Ai *et al*. [50] found in their research results on the fruit of Amomum chinensis that freeze-drying can make the fruit of Amomum chinensis have a complete glandular hair structure, which has the best color retention effect, the lowest shell breaking rate and the best flavor profile retention, but it has the longest drying time and the highest energy consumption. Therefore, in the actual production process, attention should be given to the relationship between productivity and income, and the most suitable treatment method for mulberry drying should be selected on the basis of minimizing the economic cost.

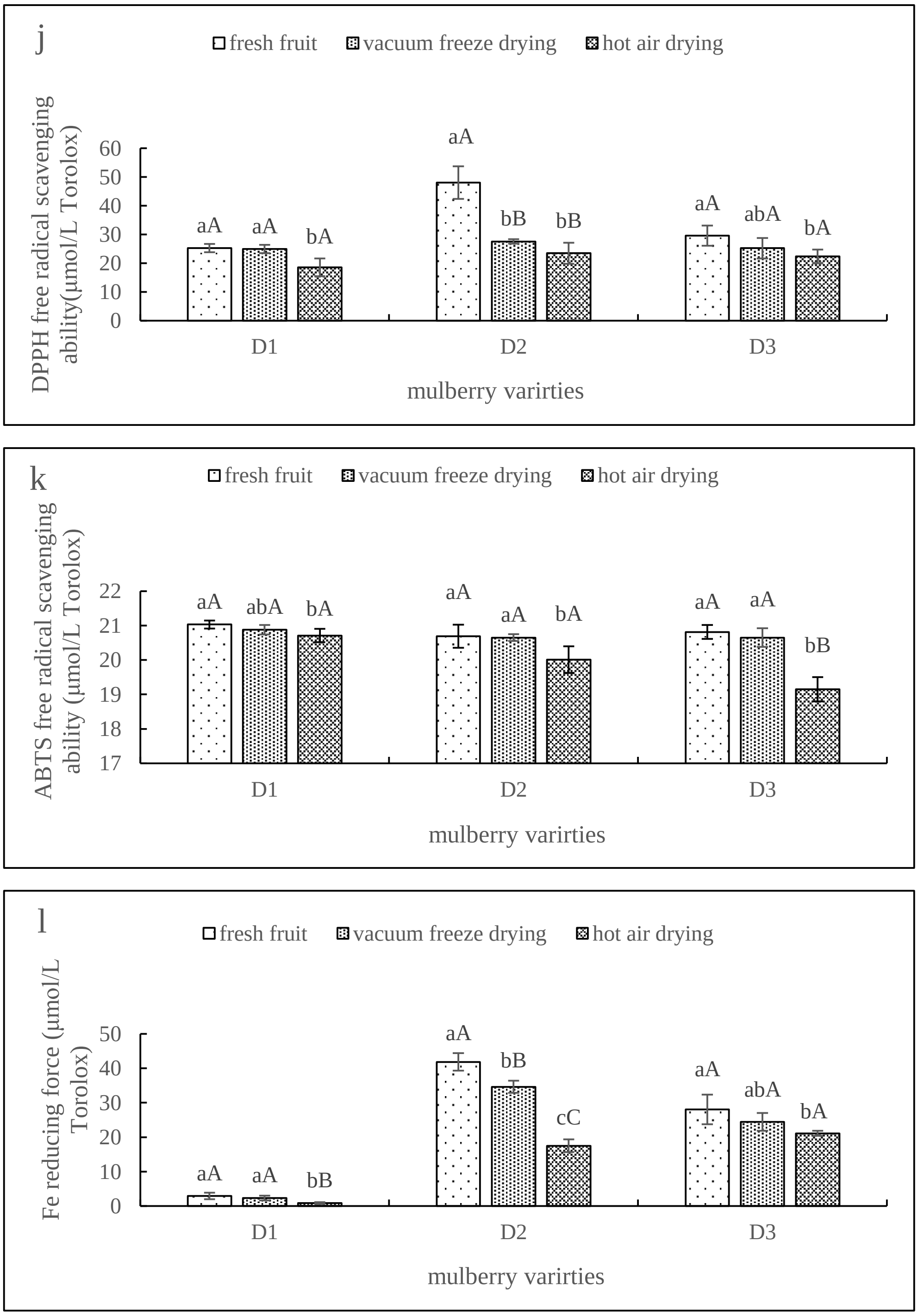

Fig. 3 (j, k, l) shows the changes in DPPH free radical scavenging ability, ABTS free radical scavenging ability and iron reducing power of fruits under different drying treatments (error bars indicate the standard error of the average). Different letters a-c in the same column indicate significant differences (P < 0.05).

4 Conclusion

In this study, it is believed that mulberry fruits are rich in nutrients, functional components and antioxidant properties, and the vacuum freeze-drying method is higher than the hot air drying method

in nutrients such as amino acid content, functional components such as polyphenols, flavonoids and antioxidant properties such as DPPH free radical scavenging ability of mulberry fruits. However, different drying methods had different effects on mulberry fruits. The contents of various indexes of dried mulberry fruits after vacuum freeze-drying and hot air drying decreased significantly compared with fresh mulberry fruits, but the loss rate of vacuum freeze-drying was the lowest, the loss rate of hot air drying was higher, and the shape and color of mulberry fruits could be better preserved by freeze-drying. Vacuum freeze-drying treatment can preserve the active ingredients in mulberry to the greatest extent and is suitable for processing various nutritional foods in the future.

Author Contributions

Investigation : Ningwei Yang

Methodology : Haichao Wen

Writing – original draft : Li Wang

Writing – review & editing: Hongjiao Li

References

1. Shadung K G, Mashela P W, Mulaudzi V L. Responses of cucurbitacin A and B concentrations from fruits of Cucumis myriocarpus and Cucumis africanus to drying method[J]. Research on Crops, 2017, 18(3): 569-573.
2. Wang H, Chen D D, Zhou H, *et al*. Molecular cloning and induced expression of the plasma membrane intrinsic protein gene and promoter from mulberry (Morus multicaulis) [J]. Canadian Journal of Plant Science, 2018, 98(6): 1245-1253.
3. Guttapalam S, Nallapu K.V. Sri. Nutrient and Qualitative Phytochemical Analysis-evaluation of Antimicrobial Activity and Development of Products with Mulberry Leaves[J]. Current Nutrition & Food Science, 2021, 17(7):708-715.
4. Hu D W, Bao T, Lu Y, *et al*. Polysaccharide from Mulberry Fruit (Morus alba L.) Protects against Palmitic-Acid-Induced Hepatocyte Lipotoxicity by Activating the Nrf2/ARE Signaling Pathway[J]. Journal of agricultural and food chemistry, 2019, 68(46): 13016-13024.
5. Chen C, Wang P P, Huang Q, *et al*. A comparison study on polysaccharides extracted from Fructus Mori using different methods: structural characterization and glucose entrapment. [J]. Food & function, 2019, 10(6): 3684-3695.
6. Zhang D Y, Wan Y, Hao J Y, *et al*. Evaluation of the alkaloid, polyphenols, and antioxidant contents of various mulberry cultivars from different planting areas in eastern China[J]. Industrial Crops & Products, 2018, 122(1): 298-307.
7. Shreelakshmi S V, Nazareth M S, Kumar S S, *et al*. Physicochemical Composition and Characterization of Bioactive Compounds of Mulberry (Morus indica L.) Fruit During Ontogeny[J]. Plant foods for human nutrition (Dordrecht, Netherlands), 2021, 76(3): 1-7.
8. Chen Y, Du F, Wang W, *et al*. Large-scale isolation of high-purity anthocyanin monomers from mulberry fruits by

combined chromatographic techniques[J]. Journal of separation science, 2017, 40(17): 3506-3512.

9. Wang W, Li X W, Bao X W, *et al*. Extraction of polysaccharides from black mulberry fruit and their effect on enhancing antioxidant activity[J]. International Journal of Biological Macromolecules, 2018, 120(1): 1420-1429.

10. Yang Y L, Yang L, Zheng H Y. Hypolipidemic and antioxidant effects of mulberry (Morus alba L.) fruit in hyperlipidemia rats[J]. Food and Chemical Toxicology, 2010, 48(8): 2374-2379.

11. Long X S, Liao S T, Wen P, *et al*. Superior hypoglycemic activity of mulberry lacking monosaccharides is accompanied by better activation of the PI3K/Akt and AMPK signaling pathways. [J]. Food & function, 2020, 11(5): 4249-4258.

12. Wan M, Li Q, Lei Q, *et al*. Polyphenols and Polysaccharides from Morus alba L. Fruit Attenuate High-Fat Diet-Induced Metabolic Syndrome Modifying the Gut Microbiota and Metabolite Profile[J]. Foods, 2022, 11(12):1818-1818.

13. Akter F, Muhury R, Sultana A, *et al*. A Comprehensive Review of Mathematical Modeling for Drying Processes of Fruits and Vegetables[J]. International Journal of Food Science, 2022, 2022:6195257-6195257.

14. Li M, Chen Y N, Geng Y L, *et al*. Convenient use of low field nuclear magnetic resonance to determine the drying kinetics and predict the quality properties of mulberries dried in hot-blast air[J]. LWT, 2020(prepublish)：110402-110402.

15. Kim H B, Kim S K, Seok Y S, *et al*. A making the method of semi-dried mulberry fruit by vacuum freezing dryer[J]. Journal of Sericultural and Entomological Science, 2015, 53(1):50-54.

16. Mahmood T, Anwar F, Afzal N, *et al*. Influence of ripening stages and drying methods on polyphenolic content and antioxidant activities of mulberry fruits[J]. Journal of Food Measurement and Characterization, 2017, 11(4): 2171-2179.

17. Ester B, Noelia B, Laura C, *et al*. Effect of Drying Process, Encapsulation, and Storage on the Survival Rates and Gastrointestinal Resistance of L. salivarius spp. salivarius Included into a Fruit Matrix[J]. Microorganisms, 2020, 8(5):654-654.

18. Tan S, Xu Y, Zhu L, *et al*. Hot Air Drying of Seabuckthorn (Hippophae rhamnoides L.) Berries: Effects of Different Pretreatment Methods on Drying Characteristics and Quality Attributes[J]. Foods, 2022, 11(22):3675-3675.

19. Jumah R Y, Tashtoush B, Shaker R R, *et al*. Manufacturing param-eters and quality characteristic of spray dried jammed[J]. Drying Technology, 2000, 18(45): 967-984.

20. Zhao XY, Yang ZB, Gai GS, *et al*. Effect of superfine grinding on properties of ginger powder[J]. Journal of Food Engineering, 2008, 91(2):217-222.

21. Hameed A M., Elkhtab E, Mostafa M.S., *et al*. Amino acids, Solubility, Bulk Density, and Water Holding Capacity of Novel Freeze-Dried Cow’ s Skimmed Milk Fermented with Potential Probiotic Lactobacillus plantarum Bu-Eg5 and Lactobacillus rhamnosus Bu-Eg6[J]. Arabian Journal of Chemistry, 2021(prepublish):103291-103291.

22. Sowbhagya H B, Suma P F, Mahadevamma S, *et al*. Spent residue from cumin - a potential source of dietary fiber[J]. Food Chemistry, 2008, 104(3):1220-1225.

23. Bradford, M.M.A. A Rapid and sensitive method for quanti-tation of microgram quantities of protein utilizing the principle of protein-dye binding. Analytical Biochemistry, 1976, 72, 248–254.

24. Candida C R, Douglas S H, Edimara S, *et al*. Comparison of techniques for determination of soluble sugars used in feed for ruminant nutrition[J]. Semina: Ciencias Agrarias, 2015,36(1):401-408.

25. Kowalska, Sylwia, Szłyk, *et al*. Simple extraction procedure for free amino acids determination in selected gluten-free flour samples[J]. European Food Research and Technology, 2021(prepublish), 1-11.

26. Habib H, Omar S K, Mohamed H S. Estimation of Rutin and Ascorbic Acid in Some Libyan Herbal Plants byRP-HPLC[J]. Medicinal & Aromatic Plants, 2016, 5(4):1-4.

27. Masturi, Alighiri D, Nuzulina K, *et al*. Optimization of condition extraction in quantification of total flavonoid content in the seeds of the Arummanis (Mangifera indica L.) mango from Indonesia[J]. Journal of Physics: Conference Series, 2019, 1321(2):22041-22041.

28. Nezam A, Al D D, Hasan N, *et al*. In vitro anti-inflammatory activity of total phenolic content of some fruit juices in syria[J]. Research Journal of Pharmacy and Technology, 2021, 14(7):3685-3688.

29. Zhu K Y, Liu W C, Ren G Y, *et al*. Comparative study on the resveratrol extraction rate and antioxidant activity of peanut treated by different drying methods[J]. Journal of Food Process Engineering, 2022, 45(4).

30. Choma I M, Olszowy M, Studziński M, *et al*. Determination of chlorogenic acid, polyphenols and antioxidants in green coffee by thin-layer chromatography, effect-directed analysis and dot blot - comparison to HPLC and spectrophotometry methods. [J]. Journal of separation science, 2019, 42(8):1542-1549.

31. Luo Y, Cai R R, Fang J L, *et al*. Total anthocyanins and cyanidin-3-O-glucoside contents and antioxidant activities of purified extracts from eight different pigmented plants[J]. Pharmacognosy Magazine, 2019, 15(60):124-129.

32. A New Colorimetric DPPH Radical Scavenging Activity Method: Comparison with Spectrophotometric Assay in Some Medicinal Plants Used in Moroccan Pharmacopoeia[J]. Pharmaceutical Fronts, 2022, 4(2).

33. Caparino O A, Tang J, Nindo C l, *et al*. Effect of drying methods on the physical properties and microstructures of mango powder[J]. Journal of Food Engineering, 2012, 111 (1):135-148.

34. Pei F, Ying S, Gao X, *et al*. Changes in nonvolatile taste components of button mushroom (*Agaricus bisporus*) during different stages of freeze drying and freeze drying combined with microwave vacuum drying [J]. Food Chemistry, 2014, 165(15):547-554.

35. Fernandes A, Barros L, Barreira J, *et al*. Effects of different processing technologies on chemical and antioxidant parameters of Macrolepiota procera wild mushroom [J]. LWT- Food Science and Technology, 2013, 54(2): 493-499.

36. Paramanandam V, Jagadeesan G, Muniyandi K, *et al*. Comparative and variability analysis of different drying methods on phytochemical, antioxidant and phenolic contents of Ficus auriculata Lour. Fruit[J]. Phytomedicine Plus, 2021(prepublish), 100075-100075.

37. Harguindeguy M, Fissore D. On the effects of freeze-drying processes on the nutritional properties of foodstuff: A review[J]. Drying Technology, 2020, 38(7):846-868.

38. Honest N. E. K, Hu Z Y, Zhao L, *et al*. Effect of Steam Blanching and Drying on Phenolic Compounds of Litchi Pericarp[J]. Molecules, 2016, 21(6)： 729-729.

39. Neungnapa R, Jian S, John S, et al. Role of endogenous and exogenous phenolics in litchi anthocyanin degradation caused by polyphenol oxidase[J]. Food Chemistry, 2009, 115(4):1253-1256.

40. Mbondo N N, Owino W O, Ambuko J, *et al*. Effect of drying methods on the retention of bioactive compounds in African eggplant. [J]. Food science & nutrition, 2018, 6(4):814-823.

41. Wu F. Effect of hot-air drying on the Content and α-glucosidase Inhibitory efficiency of Mulberry leaves Flavonoids[P]. International Conference on Medical Imaging, Sanitation and Biological Pharmacy, 2021.

42. Lou S N, Lai Y C, Huang J D. *et al*. Dying effect on flavonoid composition and antioxidant activity of immature kumquat[J]. Food Chemistry, 2015, 171:356-363.

43. Angela P, María L C, Ana H, *et al*. Effect of different drying methods on the phenolic, flavonoid and volatile compounds of Stevia rebaudiana leaves[J]. Flavor and Fragrance Journal, 2016, 31(2).

44. Tan S, Miao Y W, Zhou C B, *et al*. Effects of Hot Air Drying on Drying Kinetics and Anthocyanin Degradation of Blood-Flesh Peach[J]. Foods, 2022, 11(11):1596-1596.

45. Ozkan K, Azime, Tunckal, *et al*. Perihan. Bioaccessibility of total phenolics and antioxidant activity of melon slices dried in a heat pump drying system[J]. Journal of Food Measurement and Characterization, 2022(prepublish):1-18.

46. Zhang C H, Yu X Q, Diao Y J, *et al*. Functionalization of carboxymethyl chitosan with chlorogenic acid: preparation, characterization, and antioxidant capacity[J]. Iranian Polymer Journal, 2020(prepublish).

47. Chen T, Shuang F F, Fu Q Y, *et al*. Evaluation of the Chemical Composition and Antioxidant Activity of Mulberry (Morus alba L.) Fruits from Different Varieties in China[J]. Molecules, 2022, 27(9):2688-2688.

48. Abbaspour G Y, Kaveh M, Fatemi H, *et al*. Combined Hot Air, Microwave, and Infrared Drying of Hawthorn Fruit: Effects of Ultrasonic Pretreatment on Drying Time, Energy, Qualitative, and Bioactive Compounds' Properties[J]. Foods, 2021, 10(5):1006-1006.

49. Igor P T, Aneta W, Krzysztof L, *et al*. Influence of different drying methods on the quality of Japanese quince fruit[J]. LWT, 2019, 114(C):108416-108416.

50. Duan Z Y, Guo Y M, Wang F G. Vacuum freeze-drying rate of fruits and vegetables based on lattice boltzmann method[J]. Editorial Office of Transactions of the Chinese Society of Agricultural Engineering, 2016, 32(14):258-264.

51. Ai Z P, Mowafy S, Liu Y H. Comparative analyses of five drying techniques on drying attributes, physicochemical aspects, and flavor components of Amomum villosum fruits[J]. LWT, 2022, 154.

Cover Letter Click here to access/download;Figure;Figures.pdf

amino acid content

	fresh fruit	vacuum freeze drying	hot air drying
D1	509.01±6.42 Aa	493.02±6.42 Ab	428.02±8.63 Bc
D2	433.48±2.86 Aa	418.79±6.64 Ab	388.58±6.94 Bc
D3	576.39±8.11 Aa	556.49±5.77 Bb	486.79±8.99 Bc

soluble protein content

	fresh fruit	vacuum freeze drying	hot air drying
D1	59.92±1.66 Aa	57.35±1.54 Aa	38.57±1.14 Bb
D2	49.03±2.07 Aa	45.65±2.26 Aa	30.85±1.86 Bb
D3	70.74±2.32 Aa	69.86±1.50 Aa	55.41±1.26 Bb

ascorbic acid content

	fresh fruit	vacuum freeze drying	hot air drying
D1	16.14±3.45 Aa	14.67±0.48 Bb	8.01±0.27 Cc
D2	82.03±2.07 Aa	77.95±3.90 Ab	56.42±2.58 Bc
D3	116.13±3.75 Aa	101.26±0.98 Bb	78.86±3.39 Cc

soluble sugar content

	fresh fruit	vacuum freeze drying	hot air drying
D1	349.55±25.51 Aa	343.50±4.45 Aa	255.20±10.17 Bb
D2	439.86±1.18 Aa	380.97±8.33 Bb	278.31±16.19 Cc
D3	270.28±13.49 Aa	231.35±4.19 Bb	207.05±16.99 Bb

polyphenol content

	fresh fruit	vacuum freeze drying	hot air drying
D1	15.46±0.93 Aa	13.42±0.54 Ab	8.27±0.54 Bc
D2	138.27±6.58 Aa	116.49±6.42 Bb	89.08±6.83 Cc
D3	165.01±1.79 Aa	157.82±3.91 Bb	144.00±4.08 Bc

resveratrol content

	fresh fruit	vacuum freeze drying	hot air drying
D1	4.01±0.34 Aa	3.63±0.27 Aa	0.97±0.12 Bb
D2	20.08±2.34 Aa	19.42±1.17 Aa	11.62±0.54 Bb
D3	41.50±1.70 Aa	38.32±3.91 Ab	26.21±1.20 Bc

chlorogenic acid content

	fresh fruit	vacuum freeze drying	hot air drying
D1	35.50±2.48 Aa	15.62±0.68 Bb	7.02±0.23 Cc
D2	43.72±1.51 Aa	30.39±2.60 Bb	19.74±0.51 Cc
D3	64.03±2.83 Aa	55.67±1.61 Bb	31.65±0.76 Cc

flavone content

	fresh fruit	vacuum freeze drying	hot air drying
D1	12.56±1.04 Aa	9.63±0.52 Bb	8.80±0.88 Bb
D2	63.25±2.06 Aa	58.71±1.44 Ab	30.71±0.69 Bc
D3	113.11±6.31 Aa	110.04±7.69 Aa	42.44±4.12 Bb

anthocyanin content

	fresh fruit	vacuum freeze drying	hot air drying
D1	1.18±0.17 Aa	0.80±0.12 Aa	0.00±0.00 Bb
D2	27.72±1.96 Aa	25.19±0.38 Aa	0.89±0.48 Bb
D3	32.51±0.89 Aa	30.61±0.70 Aa	4.56±1.00 Bb

ABTS free radical scavenging ability

	fresh fruit	vacuum freeze drying	hot air drying
D1	21.03±0.12 Aa	20.88±0.14 Aab	20.71±0.19 Ab
D2	20.65±0.33 Aa	20.69±0.10 Aa	20.00±0.38 Ab
D3	20.81±0.20 Aa	20.65±0.27 Aa	19.15±0.35 Bb

DPPH free radical scavenging ability

	fresh fruit	vacuum freeze drying	hot air drying
D1	25.25±1.44 Aa	24.97±1.45 Aa	18.56±3.12 Ab
D2	48.04±5.67 Aa	27.59±0.74 Bb	23.49±3.68 Bb
D3	29.60±3.47 Aa	25.23±3.60 Aab	22.39±2.37 Ab

iron reducing force

	fresh fruit	vacuum freeze drying	hot air drying
D1	2.91±0.94 Aa	2.35±0.68 Aa	0.90±0.27 Bb
D2	41.84±2.54 Aa	34.59±1.76 Bb	17.50±1.84 Cc
D3	28.06±4.30 Aa	24.41±2.59 Aab	21.10±0.74 Ab

Click here to access/download
Supporting Information
Supporting Information.docx

Letter to editor

Click here to access/download
Other
Letter to editor.doc

NOTES: RED means DELETE

BLUE means changes based on the editors's suggestions and some we made

BLACK means the original

Effect of vacuum freeze drying and hot air drying on dried mulberry fruit quality

Li Wang[1,2], Haichao Wen[1,2], Ningwei Yang[1,2], Hongjiao Li[1,2*]

1 College of Forestry, Agricultural University of Hebei

2 Key Laboratory of Germplasm Resources of Forest Tree and Forest Protection of Hebei Province, Baoding 071000, China

* lihongjiao0103@163.com

Abstract

Two different drying methods (vacuum freeze-drying and hot-air drying) were used to dry mulberry of three varieties 'Baiyuwang'(D1), 'Longsang'(D2) and 'Zhongshen.1'(D3), and the fresh fruit of each variety was used as the control. The effects of different processing conditions on the physical characteristics, nutrients, functional components and antioxidant activity of mulberry fruit were analyzed. The results show that after different drying methods, after vacuum freeze-drying, the physical properties of dried mulberry fruit such as wettability, hygroscopic property and water retention, soluble protein, ascorbic acid and other nutrients, functional components such as polyphenols, resveratrol, chlorogenic acid and anthocyanin, and antioxidant activities such as DPPH free radical scavenging ability and ABTS free radical scavenging ability were superior to hot air drying ($P < 0.01$). It was concluded that vacuum freeze drying was more beneficial for retaining the original quality of mulberry than hot air drying. This study can provide a retaining theoretical basis for mulberry deep processing and comprehensive development and utilization.

Key words: Mulberry; Vacuum freeze drying; Hot air drying; Nutritional quality; The active ingredient

1 Introduction

Mulberry, also known as mulberry fruit, mulberry jujube, black mulberry, etc., is the mature ear of mulberry (Morus alba L.). Mulberry has high nutritional value and is rich in amino acids [1], proteins [2], vitamins [3], minerals and other nutritional elements. It is also rich in polysaccharides [4-5], alkaloids [6], flavonoids, resveratrol [7], anthocyanins [8] and other functional active ingredients, which have the medicinal value of enhancing immunity, antioxidants, regulating blood lipids and lowering blood sugar [9-11]. It is known as "the third generation of new fruit", and by the Ministry of Health as a drug and food dual-use plant resource [12].

Mulberry is a berry,that is tender and juicy, sweet and sour, and most mature in late spring and early summer with high temperature and high humidity. Due to the high water content of its fruits, it brings great difficulties in storage, transportation and preservation. In addition, it is easy to damage and rot after harvest, difficult to store, and its shelf life is short, which greatly limits the development of the mulberry industry. Therefore, dry processing of mulberry fruit is an important way to reduce fresh fruit loss and increase product value. There are relatively many drying methods available, but drying and direct drying are usually used for mulberry fruit drying at present, both of which have advantages and disadvantages. Therefore, modern high-tech food processing technology is applied to dry mulberry, product production to extend the shelf life of food [13]. It is of great significance to improve the processing level of dried mulberry products and promote the industrialization of mulberry.

An important development trend of modern food processing technology is to maintain the nutrition, color, and flavor of food to the maximum extent. Common mulberry products include mulberry wine, dried mulberry fruit, mulberry drinks, etc. Dry processing is one of the main processing forms of mulberry, which can greatly solve the problem of intolerant storage of mulberry, and the selection of drying technology and equipment has a great influence on the nutritional quality of dried products. Hot air drying is the most commonly used drying method, but the drying speed is slower and the drying time is longer [14]. Vacuum freeze-drying is a process of removing water or other solvents from frozen biological products through sublimation. It is a raw material drying method for obtaining high quality biological products, which has the characteristics of maintaining the original biological activity, color and shape, fast sample dissolution rate and low residual moisture[15].

A large number of experimental studies have been conducted on the drying characteristics of mulberry under various drying methods at home and abroad. Mahmood *et al*. [16] used natural drying and sun drying treatments for different varieties of mulberry, and the results showed that drying conditions had significant effects on the polyphenol and antioxidant properties of mulberry. The research results of Ester *et al*. [17] showed that freeze-drying treatment made samples have better texture and color. Previous studies on hot air drying and vacuum freeze-drying of mulberry have been conducted, but there is a lack of systematic studies on the effects of hot air drying and vacuum freeze-drying on the comprehensive quality of different varieties of mulberry [18]. In this study, hot air drying and vacuum freeze-drying techniques were used to explore the effects of drying methods on the physical properties, nutritional quality, functional components and antioxidant activity of dried D1, D2 and D3 of the three mulberry varieties, which were all planted in Dingzhou for 5 years, and D1 was a white variety. D2 is a northern black variety and D3 is a southern purple black variety. To provide a theoretical reference for choosing different mulberry drying treatment methods, the changes in dried fruit quality were discussed.

2. Materials and Methods

Mulberries (D1, D2 and D3) were all picked in Dongsheng Ecological Park, Dingzhou City, Hebei Province. Mulberry was picked manually and each sample was put into a separate bag after picking. The mulberry was tested immediately after being transported to the laboratory.

Vacuum freeze-drying and hot air drying are adopted.

The chemical reagents used in the whole study were of analytical grade. 2, 2'-Azino-bis(3-ethylbenzothiazoline-6-sulfonic acid), 2,2-diphenyl-1-picrylhydrazyl (EPR spectroscopy), 2,4,6-tris(2-pyridyl)-s-triazine and Folin-Ciocalteu were purchased from Solarbio, and bovine serum protein, glycine, rutin, resveratrol and chlorogenic acid standards were purchased from Sourleaf Biological Co., Ltd. Reagents such as anthrone, concentrated sulfuric acid, ninhydrin, sodium hydroxide, ethanol, sodium carbonate and sucrose were purchased from Sinopharm Chemical Reagent Co., Ltd.

2.1 Drying Process

The fresh mulberry was dried in a vacuum freeze dryer and air drying oven. Two different drying methods were used to take a fixed weight sample (150 g). All fresh mulberries were dried until they reached a constant weight. The following drying modes were used for sample preparation. All fresh mulberry fruits were dried until they reached a constant weight. The following drying mode was used for the preparation of samples.

2.1.1 Vacuum freeze-drying

Fresh mulberry (150 g) was completely frozen in a deep refrigerator at -50°C (24 hours) and then dried. Then, a vacuum freeze-dryer (FDU-1200) was used to dry for 48 h in a vacuum freeze-dryer under 20 pA at a constant temperature of -50°C.

2.1.2 Hot air drying

Fresh mulberry was spread on a perforated steel plate and dried in hot air at 60℃ for 48 h.

2.2 Physical and chemical analysis

Wettability was measured by referring to the method of Jumah *et al*. [18]. Then, 100 mL distilled water was added to a 250 mL beaker, 0.5 g mulberry powder was evenly spread on the water surface, and the time (s) required for all the powder to be wetted by water was recorded immediately. The test was repeated three times, and the average value was taken to represent the wettability of dried mulberry powder.

The moisture absorption method of a reference person such as Zhao [19] takes mulberry 3 g dried fruit powder, evenly spread in clean in a petri dish, and then puts the glass dish in an enamel disc, adding enough and saturated sodium chloride solution, after the two layers of sealing surface with plastic wrap and place for 7 days, accurate said in petri dishes and according to the formula to calculate the quality with the sample moisture absorption. The formula is as follows:

$$\text{Hygroscopicity (\%)} = \frac{\frac{m}{M+m}\times 100}{1+m/M}$$

where m is the weight change of mulberry powder before and after (g).

M: initial mass of mulberry powder (g).

The method of Hameed *et al*. [20] was used for determination by hydraulic reference. Mulberry leaf powder (0.5 g) was accurately weighed into a 10 mL centrifuge tube, 10 mL distilled water was added, thoroughly mixed for 10 min, and then centrifuged at 5000 R/min for 10 min. The supernatant was discarded, the residual water on the wall of the centrifuge tube was dried with filter paper, and

the mass was weighed. This was repeated three times, and the average value and standard deviation were used for drawing. The formula is as follows:

Hydraulic（g/g) = (M-m)/m

where M is the mass of dried fruit powder after centrifugation (g).

m: Initial mass of dried fruit powder (g).

The bulk density was measured according to the method of Sowbhagya *et al*. [21]. A certain amount of mulberry powder was put into a 10 mL centrifuge tube and shocked until the mulberry powder was just filled to the calibration line of the volumetric bottle. The bulk density (DO) of the powder was calculated by the following formula:

do (g/m L) = (M-m)/10

where M is the total weight of the fruit powder and centrifuge tube (g).

m: Test tube weight (g).

According to the method of Hameed *et al*. [22], 0.2 g of mulberry powder was placed in a centrifuge tube, 10 mL of distilled water was added, and the mixture was stirred evenly. After centrifugation at 5000 R/min for 5 min, 5 mL of the obtained supernatant was placed into a glass Petri dish, which was placed in an oven, and dried at 105°C for 3 h, and the total mass after drying was weighed. The formula is as follows:

solubility (%) = $(M-m)\times 2\times 100/m_1$

where M is the total weight of the dried Petri dish and dissolved fruit powder (g).

m: Net weight of Petri dish (g).

m_1: Initial weight of mulberry powder (g).

2.3 Nutrient content

Soluble protein was determined according to the method of Bradford [23]. The mulberry sample (0.1 g) was accurately weighed and placed in a mortar with 5.0 mL H_3PO_4 buffer (pH=7.8), and fully ground. Then the mortar was rinsed with 5.0 mL of the above buffer and poured into a 10.0 mL centrifuge tube. The samples were centrifuged at a constant temperature of 10000 RMPs for 20 min at 4°C. Then, 1.0 mL of the supernatant was aspirated, 5.0 mL of Coomassie brilliant blue G-250 solution was added, and the absorbance value was measured at 595 nm after 2 min of mixing.

The soluble sugar content was determined according to the method of Candida [24].The mulberry sample of 0.1 g was accurately weighed and ground thoroughly with 10.0 mL distilled water in a mortar, placed in a 10 mL centrifuge tube, sealed and heated in a boiling water bath for 30 min.The heated solution was filtered through gauze, the rinsed residue was filtered repeatedly with distilled water, and the volume was stabilized into a 25 mL volumetric flask. Then, 1.5 mL of distilled water,

0.5 mL of anthrone, ethyl acetate reagent and 5.0 mL of concentrated sulfuric acid were added to the test tube in sequence and mixed with shock. The absorbance value was measured at 630 nm wavelength after the sample was cooled to room temperature. Finally, the soluble sugar content of the samples was calculated according to the standard curve and formula.

The content of free amino acids was determined by reference to the ninhydrin chromogenic method [25]. First, the mulberry sample was weighed by an analytical balance and ground into a slurry in a mortar. Then, 5.0 mL distilled water was added to the mortar and placed into the centrifuge tube. Then 5.0 mL distilled water was added to rinse the mortar and poured into the centrifuge tube. Ultrasonic extraction was carried out at 40°C for 20 min using an ultrasonic cleaning machine. After extraction, it was centrifuged at 5000 rpm for 25 min, and 1.0 mL of the supernatant was absorbed by a pipetting nozzle, Then, 2.0 mL of H_3PO_4 buffer with pH 6.0 was added, and 3.0 mL of ninhydrin solution was added after full shaking and standing for 5 min. The centrifuge tube was sealed and heated in a boiling water bath for 30 min. After cooling, the absorbance was measured at 560 nm, and the amino acid content was calculated according to the regression equation.

Determination of ascorbic acid (VC) content: The method of Habib *et al.* [26] was used for determination.

2.4 Content of functional components

The content of flavonoids in mulberry was determined by the $AlCl_3$ chromogenic method [27]. First, a 0.2 g mulberry sample was accurately weighed by an analytical balance, and 5.0 mL 70% C_2H_5OH was added according to a material to liquid ratio of 1:25, and placed into a centrifuge tube. Then, an ultrasonic cleaning machine was used for extraction for 40 min at a constant temperature of 25 ℃. The extracted extract was centrifuged at 7000 RPM for 10 min. For determination, 1.0 mL of supernatant was taken, 0.4 mL of 50% $NaNO_3$ solution and 0.4 mL of 10% $AlCl_3$ solution were added, the mixture was thoroughly shaken and left for 5 min, and 4 mL of 4% NaOH solution was added. A constant volume of 70% C_2H_5OH was used to 10.0 mL. After standing for 10 min at room temperature, the absorbance value was measured at a wavelength of 415 nm. Finally, the content of flavonoids in the samples was calculated according to the standard curve.

The content of polyphenols was determined by the Folin-Ciocalteu colorimetric method [28]. First, a 0.1 g mulberry sample was accurately weighed by an analytical balance, and 10.0 ml 50% C_2H_5OH was added according to a solid-liquid ratio of 1:100. The samples were extracted by ultrasonication for 40 min at a constant temperature of 25°C. This was followed by centrifugation at 7000 rpm for 10 min. After centrifugation, 1.0 mL of the supernatant was taken, 1.0 mL of folinol reagent was added, thoroughly shaken and mixed, and the mixture was allowed to stand for 5 min at

room temperature. Then, 4.0 ml 20% $NACO_3$ solution was added and heated in a 50°C water bath for 1 hour. After cooling to room temperature, the absorbance value was measured at 760 nm wavelength. Finally, the content of polyphenols in the samples was calculated according to the standard curve regression equation.

The resveratrol content was determined by the C_2H_5OH extraction method [29]. A mulberry sample of 0.2 g was accurately weighed, and 60% C_2H_5OH (3.0 mL) was added according to a solid-liquid ratio of 1:15, and placed into a 10.0 mL centrifuge tube. After shaking well, the mulberry sample was heated in a water bath at 60°C for 1 h, and the mulberry sample was kept away from light during the whole process. After filtration, samples were centrifuged at 7000 rpm for 10 min. For determination, 0.5 mL of supernatant was diluted 10 times with the above concentration of C_2H_5OH, the absorbance value was measured at 305 nm wavelength, and the content of resveratrol was calculated according to the standard curve and regression equation.

The chlorogenic acid content was determined by the Fe2+ chromogenic method [30].The 0.2 g sample was accurately weighed, and 10.0 ml 60% CH_3OH was added as the extraction agent according to the solid-liquid ratio of 1:5. The samples were extracted by ultrasound for 30 min.After simple filtration, 60% CH_3OH was used to a constant volume of 15.0 mL, 5.0 mL of constant volume extract was taken, 0.5 mL of 0.2 mol/L $FeCl_3$ solution was added, and the mixture was shaken and left for 60 min.The absorbance value was measured at a wavelength of 755 nm, and the chlorogenic acid content was calculated according to the standard curve and regression equation.

The anthocyanin content in mulberry was determined by the pH difference method [31]. A 0.2 g mulberry sample was accurately weighed by an analytical balance, 4.0 ml of 60% acidified C_2H_5OH was added according to a 1:20 solid-liquid ratio, and the whole process was kept away from light. Then the samples were placed in a 60°C water bath and heated for 2 h. The samples were allowed to cool to room temperature. Then 1.0 mL supernatant and 9.0 mL H_3PO_4 buffer with pH=1.0 and pH=4.5 were added to two 25 mL vials, shaken well, and left in the dark for 2 hours. The absorbance values were measured at wavelengths of 520 nm and 700 nm.

2.5 Antioxidant properties

DPPH free radical scavenging ability, ABTS free radical scavenging ability and iron reducing power of mulberry [32].

The DPPH radical scavenging ability was determined by the DPPH radical scavenging method. A mulberry sample of 0.1 g was accurately weighed by an analytical balance, and 50% C_2H_5OH (0.5 mL) was added according to a solid-liquid ratio of 1:50. After ultrasonic extraction, the volume was fixed to 10.0 mL for later use. For measurement, 0.5 mL of the extract was taken, 2.0 mL of DPPH

reaction solution was added, and the reaction was shaken and kept away from light for 30 min. The light absorption value was measured at a wavelength of 517 nm. In the control group, the same volume of CH_3OH was used instead of the extract, and the final results were expressed as μmol/LTrolox equivalent antioxidant capacity. The measured absorbance value was taken as the abscissa, and the concentration of Trolox standard solution was taken as the ordinate. The standard curve was drawn, and the regression equation was calculated.

In the determination of ABTS free radical scavenging ability, the method of preparing the extract was the same as that of DPPH, and ABTS+ working solution was prepared in advance:10.0 ml ABTS solution of 7 mmol/L and 5.0 potassium persulfate solution of 7.35 mmol/L were mixed, placed in the dark for 16 h at room temperature, and then prepared for use. The absorbance was diluted to 0.70 ± 0.02 at 734 nm when used. At the time of measurement, 0.5 mL of the extract was taken, 2.0 mL ABTS+ working solution was added, and the reaction was kept away from light for 6 min after shaking. The absorbance value was measured at a wavelength of 734 nm. The final results were expressed as μmol/LTrolox equivalent antioxidant capacity. The measured absorbance value is the abscissa, the concentration of Trolox standard solution is the ordinate, the standard curve is drawn, and the regression equation is calculated.

Iron reducing capacity was determined by the FRAP method. The method of preparing the extract in the determination is the same as that in DPPH. Before the test, the TPTZ working solution was prepared in advance: CH_3COONa solution pH=3.6. Then, 300 mmol/L, 10 mmol/L, TPTZ solution, and 20 mmol/L, $FeCL_3 \cdot 6H_2O$ solution were mixed according to a volume ratio of 1:1:1. For measurement, 0.5 mL of extract was taken, 1.0 mL of distilled water and 1.8 mL of TPTZ working solution were added, and the reaction was kept away from light for 10 min. The absorbance value was measured at a wavelength of 593 nm, and the final result was expressed as the antioxidant capacity of μmol/L Trolox. The measured absorbance value is the abscissa, and the concentration of Trolox standard solution is the ordinate. The standard curve was drawn and the regression equation was calculated.

2.6 Statistical Analysis

A completely randomized design (CRD) was used to analyze the physicochemical properties, nutrients, functional components and antioxidant properties of mulberry fruit. One-way analysis of variance (P <0.05) was used to analyze significant differences between treatments. The parameters of chemical and antioxidant properties of different varieties of mulberry were repeated three times in these studies.

3 Results and discussion

Table 1 shows the physical properties of mulberry fruit under different drying modes (vacuum freeze-drying and hot-air drying). Figure 1 shows the changes in the nutrient composition of mulberry fruit under different drying modes. Figure 2 shows the changes in the functional components of mulberry fruit under different drying modes. Figure 3 shows the variation in the antioxidant capacity of mulberry fruit under different drying modes.

3.1 Changes in physical characteristics under different drying treatments

Table 1 describes the effects of the different drying processes used in this study on fruit wettability, hygroscopicity, water retention, bulk density and solubility. In terms of physical and chemical properties, fruit wettability, moisture absorption, water retention, bulk density and solubility all reflect the effects of drying processes on fruit quality. The results in Table 1 show that the physicochemical properties of different mulberry varieties after vacuum freeze-drying were better than those after hot air drying, which indicated that the use of exhaust heat from the condenser as the heat source could significantly accelerate the rate of water content decline. These data indicate that an important feature of vacuum freeze dryers is the efficient use of energy to reduce moisture content compared to other drying methods.

Moisture content is a very important characteristic of freeze-dried foods, as it affects the appearance, texture and taste of the product. Moisture content also affects the freshness and shelf life of products. A high moisture content will make it easy for bacteria, mold and yeast to multiply, and the food will change. The vacuum freeze-drying process removes moisture from the sample. In terms of the physical properties of mulberry, the fruit powder with packing density, hygroscopic property, wettability and solubility after vacuum freezing is better than that after hot air drying, which is related to the hardening phenomenon on the surface of the mulberry powder after hot air drying and the inability to form a large number of micropores inside the fruit powder, which is consistent with the results of Caparino [33].

Table 1

Effects of different drying methods on the physical properties of mulberry fruit powder

Varieties	process mode	Wettability(s)	Hygroscopicity(%)	water-holding power(g/g)	bulk density(g/mL)	Solubleness(%)
D1	vacuum freeze drying	241.00±22.07aA	9.13±1.59aA	2.09±0.05aA	0.46±0.07aA	28.15±14.68aA
	hot air drying	55.00±8.50bB	4.69±2.90bB	1.69±0.33aA	0.53±0.14bB	35.03±0.84aA
D2	vacuum freeze drying	65.00±7.23aA	10.41±0.47aA	2.09±0.09aA	0.54±0.09aA	38.41±2.50aA

	hot air drying	27.00±5.86bB	7.74±0.72bB	1.38±0.02bB	0.52±0.07aA	41.57±2.41aA
D3	vacuum freeze drying	144.00±20.60aA	10.35±0.68aA	2.17±0.01aA	0.47±0.21aA	37.75±0.81aA
	hot air drying	110.00±10.50aA	7.39±0.82bB	1.24±0.00bB	0.66±0.59bB	43.48±9.74aA

Note: Lowercase letters indicate the significance of differences between different treatments of the same variety (P<0.05), uppercase letters indicate the significance of differences between different treatments of the same variety (P<0.01).

3.2 Changes in nutrient content under different drying treatments

The results of Figure 1 show that after vacuum freeze-drying and hot-air drying, the nutrient content of mulberry fruits of the three varieties changed. Compared with fresh mulberry fruit, the content of mulberry fruit showed a decreasing trend after different drying treatments, but the retention of these components by vacuum freeze-drying was better than that by hot air drying, which may be because hot air drying would promote the Maillard reaction and Strecker degradation reaction of amino acids in mulberry fruit, resulting in the loss of amino acids [34].The reason why vacuum freeze-drying reduces the content of amino acids may be because freezing can prevent proteins from degrading into amino acids [35]. The research results of Paramanandam *et al*. [36] found that the content of amino acids and proteins in fruits under hot air drying was higher, which is different from the research results in this paper and needs to be further verified. In addition, the research results of Harguindeguy *et al*. [37] show that different drying processes have an impact on the ascorbic acid content, phenolic compounds and antioxidant properties of food.

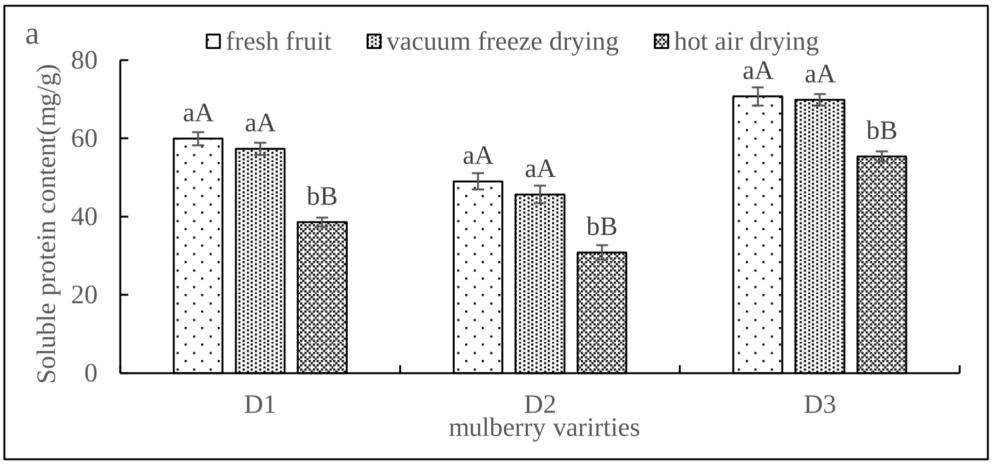

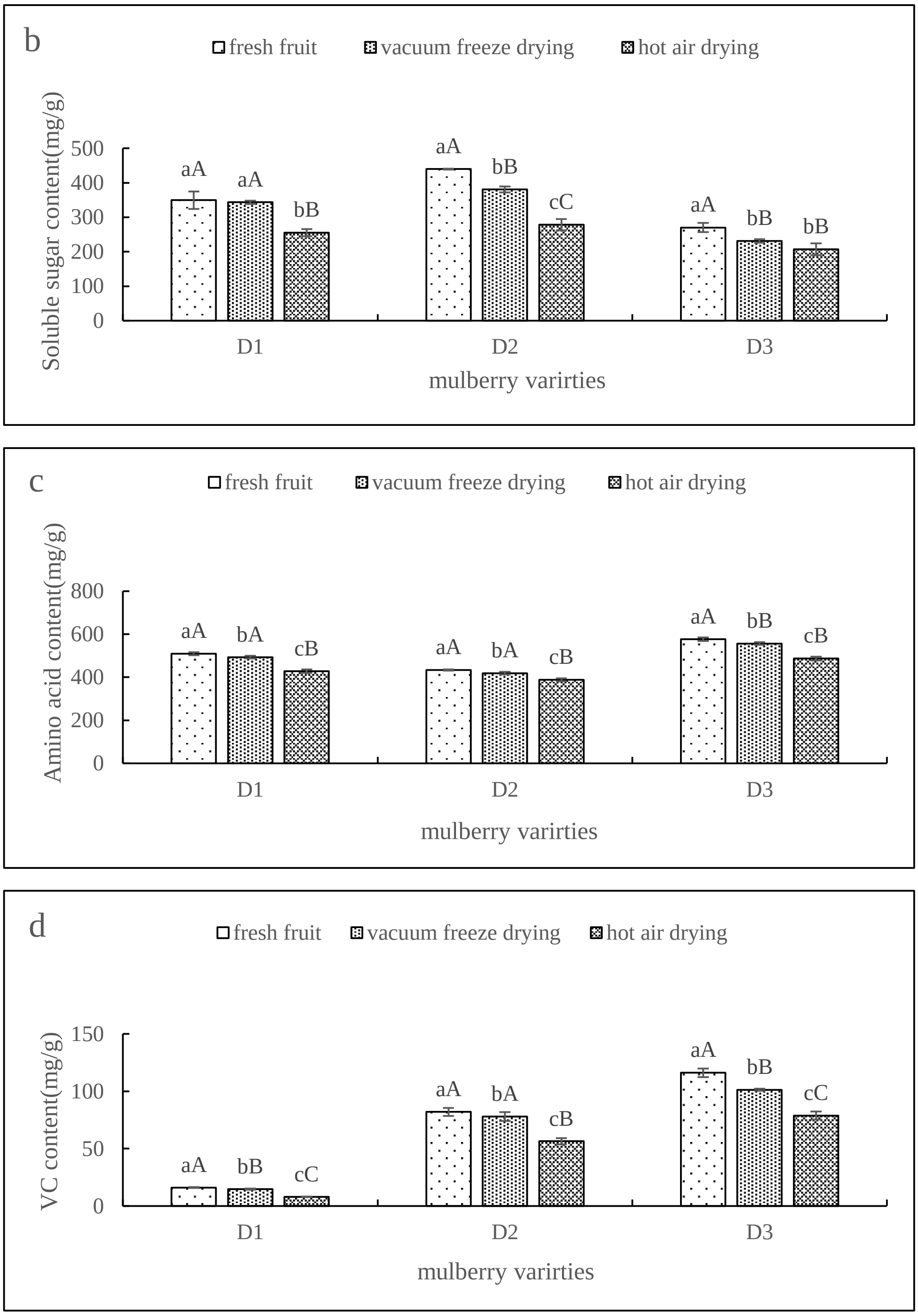

Fig.1 (a, b, c, d) shows the changes in soluble protein, soluble sugar, amino acid and ascorbic acid contents of fruits under different drying treatments (error bars indicate the standard error of the average). Different letters a-c in the same column indicate significant differences ($P < 0.05$).

3.3 Content changes of functional components under different drying treatments

Figure. 2 shows the changes in the functional components of the three mulberry fruits under different drying modes. According to the data, compared with fresh mulberry fruit, the contents of total phenols, total flavonoids, ascorbic acid and neo-chlorogenic acid under vacuum freeze-drying

were significantly higher than those under hot air drying after different drying treatments. This was because the functional components such as polyphenols, flavonoids and vitamins in mulberry were affected by light, temperature, oxygen partial pressure, water activity and other factors during the drying process, and oxidation, aggregation or decomposition occurred, resulting in changes in the content of functional components [38-40]. In addition, under the conditions of low temperature and low oxygen partial pressure, the activity of oxidase is low. Therefore, thermally sensitive components such as polyphenols, flavonoids and ascorbic acid easily undergo enzymatic oxidation and have better retention under this dry condition. Hot air drying destroys heat sensitive components, such as flavonoids [41-42]. The research results of Angela *et al*. [43] showed that compared with hot-air drying, the content of flavonoids and phenolic acids was higher under freeze-drying. Tan *et al*. [44] found that the content of anthocyanins in fresh fruits was the highest and decreased with increasing drying temperature. According to the analysis, cyanidin-3-O-glucoside and delphinidin-3-O-galactoside were the main anthocyanins in flesh-and-blood peaches, but during the whole drying process, the content of delphinidin-3-O-galactoside decreased continuously, which was consistent with the results of this study.

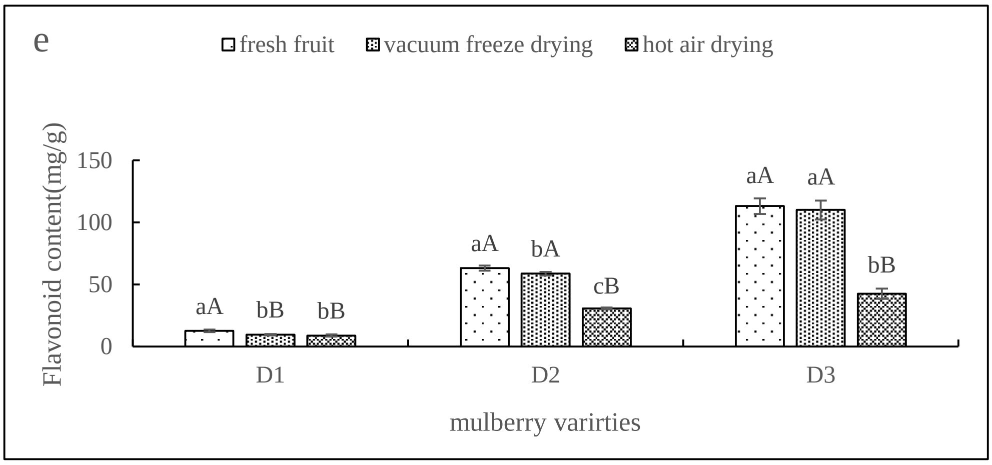

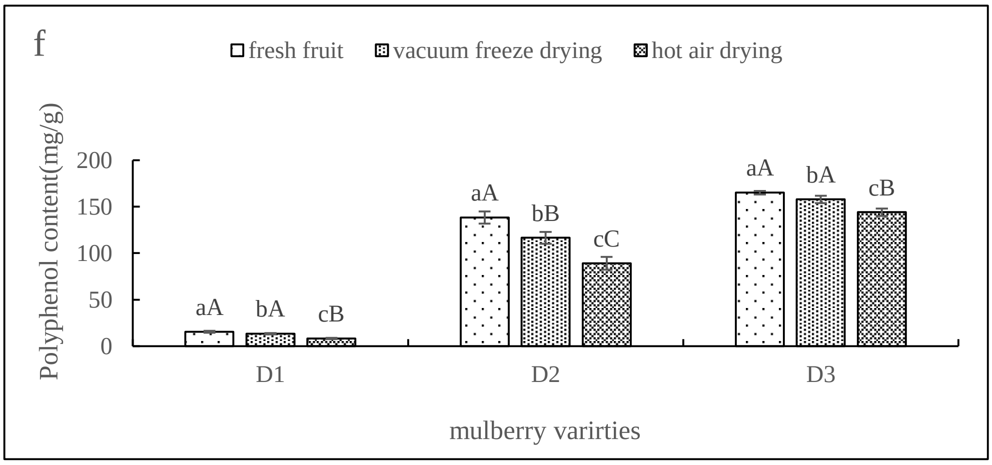

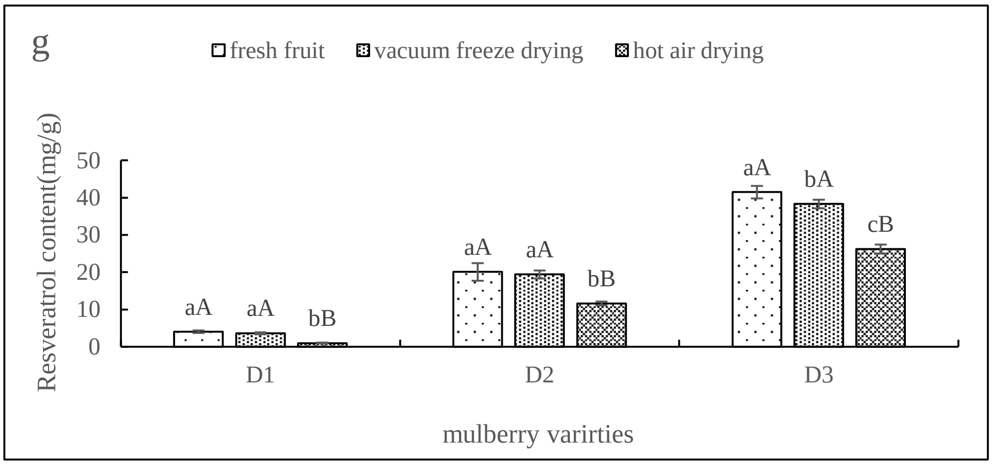

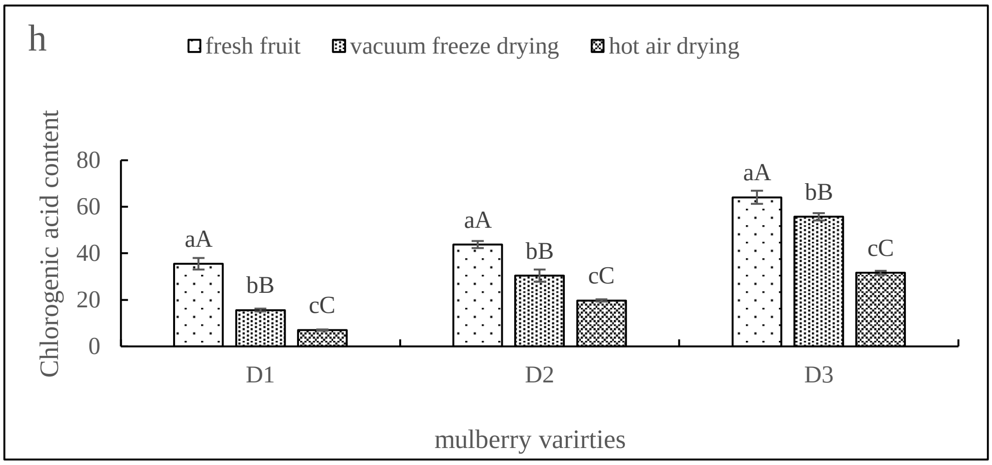

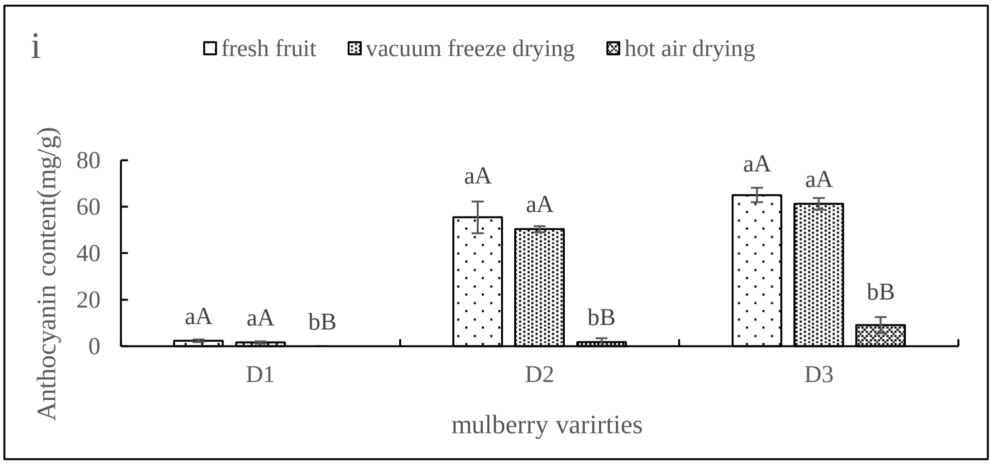

Fig 2 (e, f, g, h) shows changes in the contents of flavonoids, polyphenols, resveratrol, chlorogenic acid and anthocyanins in fruits under different drying treatments (error bars indicate the standard error of the average). Different letters a-c in the same column indicate significant differences ($P < 0.05$).

3.4 Changes in antioxidant properties under different drying treatments

Total phenols and flavonoids were the main functional components of mulberry, and phenolic compounds composed of chlorogenic acid and its derivatives were dominant. Polyphenol compounds contain multiple phenolic hydroxyl groups and have strong reducing properties. Their structural

properties are the main reason for the antioxidant activity of these compounds. They achieve antioxidant activities mainly by directly scavenging free radicals or interacting with metal ions. The results showed that the antioxidant activity of mulberry under vacuum freeze-drying was stronger, which might be due to the better retention and reducibility of phenolic compounds under vacuum freeze-drying. Ozkan *et al*. [45] reported a similar trend in their findings. This was also confirmed by the results of Zhang *et al*. [46], who found that the scavenging activity of chlorogenic acid on DPPH and ABTS free radicals reached 92.4% and 99.4% respectively. Chen *et al*. [47] determined the total phenols, flavonoids, vitamin C and antioxidant capacity of 25 mulberry samples from 6 provinces, and the results showed that D3 had the highest health care value and the highest active compound.content The data in the same figure show that the antioxidant activity of mulberry under vacuum freeze-drying is high, while that under hot-air drying is low. Therefore, the contents of total phenols, flavonoids and antioxidant substances in mulberry have great influence on the antioxidant capacity of mulberry, and the contents of functional components in mulberry fruits, especially phenolic substances, are affected by different drying methods. The drying method also determines its oxidation resistance.

In conclusion, both hot air drying and vacuum freeze-drying had significant effects on the dry quality characteristics of mulberry fruit, and vacuum freeze-drying could more effectively retain the nutritional quality, functional components and antioxidant activity of fresh mulberry fruit. This study provides an effective reference for the further development and utilization of mulberry products.It can also provide some reference for further study on the effect of drying methods on mulberry fruit drying.Abbaspour, Igor and Duan *et al*. [47-49] stated that freeze-drying has the lowest color and shrinkage rate of fruits, the highest content of bioactive compounds, and better rehydration performance, and can maintain the original quality to the maximum extent, so it has broader application prospects in food, medicine, biological products and other fields. However, the relationship between the drying method and fruit characteristics should be considered at the same time. Ai *et al*. [50] found in their research results on the fruit of Amomum chinensis that freeze-drying can make the fruit of Amomum chinensis have a complete glandular hair structure, which has the best color retention effect, the lowest shell breaking rate and the best flavor profile retention, but it has the longest drying time and the highest energy consumption. Therefore, in the actual production process, attention should be given to the relationship between productivity and income, and the most suitable treatment method for mulberry drying should be selected on the basis of minimizing the economic cost.

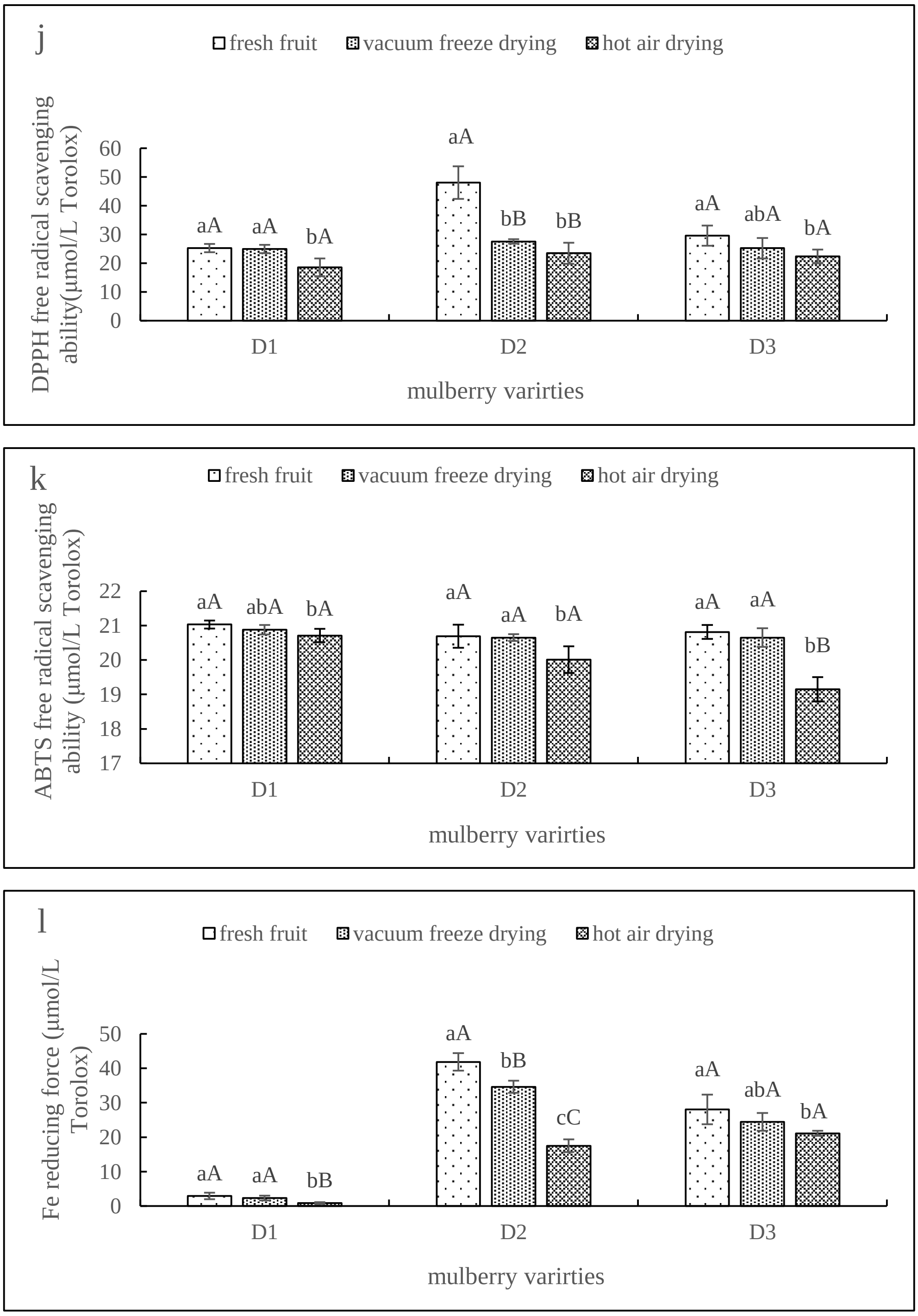

Fig. 3 (j, k, l) shows the changes in DPPH free radical scavenging ability, ABTS free radical scavenging ability and iron reducing power of fruits under different drying treatments (error bars indicate the standard error of the average). Different letters a-c in the same column indicate significant differences ($P < 0.05$).

4 Conclusion

In this study, it is believed that mulberry fruits are rich in nutrients, functional components and antioxidant properties, and the vacuum freeze-drying method is higher than the hot air drying method

in nutrients such as amino acid content, functional components such as polyphenols, flavonoids and antioxidant properties such as DPPH free radical scavenging ability of mulberry fruits. However, different drying methods had different effects on mulberry fruits. The contents of various indexes of dried mulberry fruits after vacuum freeze-drying and hot air drying decreased significantly compared with fresh mulberry fruits, but the loss rate of vacuum freeze-drying was the lowest, the loss rate of hot air drying was higher, and the shape and color of mulberry fruits could be better preserved by freeze-drying. Vacuum freeze-drying treatment can preserve the active ingredients in mulberry to the greatest extent and is suitable for processing various nutritional foods in the future.

Author Contributions

Investigation : Ningwei Yang

Methodology : Haichao Wen

Writing – original draft : Li Wang

Writing – review & editing: Hongjiao Li

References

1. Shadung K G, Mashela P W, Mulaudzi V L. Responses of cucurbitacin A and B concentrations from fruits of Cucumis myriocarpus and Cucumis africanus to drying method[J]. Research on Crops, 2017, 18(3): 569-573.
2. Wang H, Chen D D, Zhou H, *et al*. Molecular cloning and induced expression of the plasma membrane intrinsic protein gene and promoter from mulberry (Morus multicaulis) [J]. Canadian Journal of Plant Science, 2018, 98(6): 1245-1253.
3. Guttapalam S, Nallapu K.V. Sri. Nutrient and Qualitative Phytochemical Analysis-evaluation of Antimicrobial Activity and Development of Products with Mulberry Leaves[J]. Current Nutrition & Food Science, 2021, 17(7):708-715.
4. Hu D W, Bao T, Lu Y, *et al*. Polysaccharide from Mulberry Fruit (Morus alba L.) Protects against Palmitic-Acid-Induced Hepatocyte Lipotoxicity by Activating the Nrf2/ARE Signaling Pathway[J]. Journal of agricultural and food chemistry, 2019, 68(46): 13016-13024.
5. Chen C, Wang P P, Huang Q, *et al*. A comparison study on polysaccharides extracted from Fructus Mori using different methods: structural characterization and glucose entrapment. [J]. Food & function, 2019, 10(6): 3684-3695.
6. Zhang D Y, Wan Y, Hao J Y, *et al*. Evaluation of the alkaloid, polyphenols, and antioxidant contents of various mulberry cultivars from different planting areas in eastern China[J]. Industrial Crops & Products, 2018, 122(1): 298-307.
7. Shreelakshmi S V, Nazareth M S, Kumar S S, *et al*. Physicochemical Composition and Characterization of Bioactive Compounds of Mulberry (Morus indica L.) Fruit During Ontogeny[J]. Plant foods for human nutrition (Dordrecht, Netherlands), 2021, 76(3): 1-7.
8. Chen Y, Du F, Wang W, *et al*. Large-scale isolation of high-purity anthocyanin monomers from mulberry fruits by

combined chromatographic techniques[J]. Journal of separation science, 2017, 40(17): 3506-3512.

9. Wang W, Li X W, Bao X W, *et al*. Extraction of polysaccharides from black mulberry fruit and their effect on enhancing antioxidant activity[J]. International Journal of Biological Macromolecules, 2018, 120(1): 1420-1429.

10. Yang Y L, Yang L, Zheng H Y. Hypolipidemic and antioxidant effects of mulberry (Morus alba L.) fruit in hyperlipidemia rats[J]. Food and Chemical Toxicology, 2010, 48(8): 2374-2379.

11. Long X S, Liao S T, Wen P, *et al*. Superior hypoglycemic activity of mulberry lacking monosaccharides is accompanied by better activation of the PI3K/Akt and AMPK signaling pathways. [J]. Food & function, 2020, 11(5): 4249-4258.

12. Wan M, Li Q, Lei Q, *et al*. Polyphenols and Polysaccharides from Morus alba L. Fruit Attenuate High-Fat Diet-Induced Metabolic Syndrome Modifying the Gut Microbiota and Metabolite Profile[J]. Foods, 2022, 11(12):1818-1818.

13. Akter F, Muhury R, Sultana A, *et al*. A Comprehensive Review of Mathematical Modeling for Drying Processes of Fruits and Vegetables[J]. International Journal of Food Science, 2022, 2022:6195257-6195257.

14. Li M, Chen Y N, Geng Y L, *et al*. Convenient use of low field nuclear magnetic resonance to determine the drying kinetics and predict the quality properties of mulberries dried in hot-blast air[J]. LWT, 2020(prepublish): 110402-110402.

15. Kim H B, Kim S K, Seok Y S, *et al*. A making the method of semi-dried mulberry fruit by vacuum freezing dryer[J]. Journal of Sericultural and Entomological Science, 2015, 53(1):50-54.

16. Mahmood T, Anwar F, Afzal N, *et al*. Influence of ripening stages and drying methods on polyphenolic content and antioxidant activities of mulberry fruits[J]. Journal of Food Measurement and Characterization, 2017, 11(4): 2171-2179.

17. Ester B, Noelia B, Laura C, *et al*. Effect of Drying Process, Encapsulation, and Storage on the Survival Rates and Gastrointestinal Resistance of L. salivarius spp. salivarius Included into a Fruit Matrix[J]. Microorganisms, 2020, 8(5):654-654.

18. Tan S, Xu Y, Zhu L, *et al*. Hot Air Drying of Seabuckthorn (Hippophae rhamnoides L.) Berries: Effects of Different Pretreatment Methods on Drying Characteristics and Quality Attributes[J]. Foods, 2022, 11(22):3675-3675.

19. Jumah R Y, Tashtoush B, Shaker R R, *et al*. Manufacturing param-eters and quality characteristic of spray dried jammed[J]. Drying Technology, 2000, 18(45): 967-984.

20. Zhao XY, Yang ZB, Gai GS, *et al*. Effect of superfine grinding on properties of ginger powder[J]. Journal of Food Engineering, 2008, 91(2):217-222.

21. Hameed A M., Elkhtab E, Mostafa M.S., *et al*. Amino acids, Solubility, Bulk Density, and Water Holding Capacity of Novel Freeze-Dried Cow’s Skimmed Milk Fermented with Potential Probiotic Lactobacillus plantarum Bu-Eg5 and Lactobacillus rhamnosus Bu-Eg6[J]. Arabian Journal of Chemistry, 2021(prepublish):103291-103291.

22. Sowbhagya H B, Suma P F, Mahadevamma S, *et al*. Spent residue from cumin - a potential source of dietary fiber[J]. Food Chemistry, 2008, 104(3):1220-1225.

23. Bradford, M.M.A. A Rapid and sensitive method for quanti-tation of microgram quantities of protein utilizing the principle of protein-dye binding. Analytical Biochemistry, 1976, 72, 248–254.

24. Candida C R, Douglas S H, Edimara S, *et al*. Comparison of techniques for determination of soluble sugars used in feed for ruminant nutrition[J]. Semina: Ciencias Agrarias, 2015,36(1):401-408.

25. Kowalska, Sylwia, Szłyk, *et al*. Simple extraction procedure for free amino acids determination in selected gluten-free flour samples[J]. European Food Research and Technology, 2021(prepublish), 1-11.

26. Habib H, Omar S K, Mohamed H S. Estimation of Rutin and Ascorbic Acid in Some Libyan Herbal Plants byRP-HPLC[J]. Medicinal & Aromatic Plants, 2016, 5(4):1-4.

27. Masturi, Alighiri D, Nuzulina K, *et al*. Optimization of condition extraction in quantification of total flavonoid content in the seeds of the Arummanis (Mangifera indica L.) mango from Indonesia[J]. Journal of Physics: Conference Series, 2019, 1321(2):22041-22041.

28. Nezam A, Al D D, Hasan N, *et al*. In vitro anti-inflammatory activity of total phenolic content of some fruit juices in syria[J]. Research Journal of Pharmacy and Technology, 2021, 14(7):3685-3688.

29. Zhu K Y, Liu W C, Ren G Y, *et al*. Comparative study on the resveratrol extraction rate and antioxidant activity of peanut treated by different drying methods[J]. Journal of Food Process Engineering, 2022, 45(4).

30. Choma I M, Olszowy M, Studziński M, *et al*. Determination of chlorogenic acid, polyphenols and antioxidants in green coffee by thin-layer chromatography, effect-directed analysis and dot blot - comparison to HPLC and spectrophotometry methods. [J]. Journal of separation science, 2019, 42(8):1542-1549.

31. Luo Y, Cai R R, Fang J L, *et al*. Total anthocyanins and cyanidin-3-O-glucoside contents and antioxidant activities of purified extracts from eight different pigmented plants[J]. Pharmacognosy Magazine, 2019, 15(60):124-129.

32. A New Colorimetric DPPH Radical Scavenging Activity Method: Comparison with Spectrophotometric Assay in Some Medicinal Plants Used in Moroccan Pharmacopoeia[J]. Pharmaceutical Fronts, 2022, 4(2).

33. Caparino O A, Tang J, Nindo C l, *et al*. Effect of drying methods on the physical properties and microstructures of mango powder[J]. Journal of Food Engineering, 2012, 111 (1):135-148.

34. Pei F, Ying S, Gao X, *et al*. Changes in nonvolatile taste components of button mushroom (*Agaricus bisporus*) during different stages of freeze drying and freeze drying combined with microwave vacuum drying [J]. Food Chemistry, 2014, 165(15):547-554.

35. Fernandes A, Barros L, Barreira J, *et al*. Effects of different processing technologies on chemical and antioxidant parameters of Macrolepiota procera wild mushroom [J]. LWT- Food Science and Technology, 2013, 54(2): 493-499.

36. Paramanandam V, Jagadeesan G, Muniyandi K, *et al*. Comparative and variability analysis of different drying methods on phytochemical, antioxidant and phenolic contents of Ficus auriculata Lour. Fruit[J]. Phytomedicine Plus, 2021(prepublish), 100075-100075.

37. Harguindeguy M, Fissore D. On the effects of freeze-drying processes on the nutritional properties of foodstuff: A review[J]. Drying Technology, 2020, 38(7):846-868.

38. Honest N. E. K, Hu Z Y, Zhao L, *et al*. Effect of Steam Blanching and Drying on Phenolic Compounds of Litchi Pericarp[J]. Molecules, 2016, 21(6)： 729-729.

39. Neungnapa R, Jian S, John S, et al. Role of endogenous and exogenous phenolics in litchi anthocyanin degradation caused by polyphenol oxidase[J]. Food Chemistry, 2009, 115(4):1253-1256.

40. Mbondo N N, Owino W O, Ambuko J, *et al*. Effect of drying methods on the retention of bioactive compounds in African eggplant. [J]. Food science & nutrition, 2018, 6(4):814-823.

41. Wu F. Effect of hot-air drying on the Content and α-glucosidase Inhibitory efficiency of Mulberry leaves Flavonoids[P]. International Conference on Medical Imaging, Sanitation and Biological Pharmacy, 2021.

42. Lou S N, Lai Y C, Huang J D. *et al*. Dying effect on flavonoid composition and antioxidant activity of immature kumquat[J]. Food Chemistry, 2015, 171:356-363.

43. Angela P, María L C, Ana H, *et al*. Effect of different drying methods on the phenolic, flavonoid and volatile compounds of Stevia rebaudiana leaves[J]. Flavor and Fragrance Journal, 2016, 31(2).

44. Tan S, Miao Y W, Zhou C B, *et al*. Effects of Hot Air Drying on Drying Kinetics and Anthocyanin Degradation of Blood-Flesh Peach[J]. Foods, 2022, 11(11):1596-1596.

45. Ozkan K, Azime, Tunckal, *et al*. Perihan. Bioaccessibility of total phenolics and antioxidant activity of melon slices dried in a heat pump drying system[J]. Journal of Food Measurement and Characterization, 2022(prepublish):1-18.

46. Zhang C H, Yu X Q, Diao Y J, *et al*. Functionalization of carboxymethyl chitosan with chlorogenic acid: preparation, characterization, and antioxidant capacity[J]. Iranian Polymer Journal, 2020(prepublish).

47. Chen T, Shuang F F, Fu Q Y, *et al*. Evaluation of the Chemical Composition and Antioxidant Activity of Mulberry (Morus alba L.) Fruits from Different Varieties in China[J]. Molecules, 2022, 27(9):2688-2688.

48. Abbaspour G Y, Kaveh M, Fatemi H, *et al*. Combined Hot Air, Microwave, and Infrared Drying of Hawthorn Fruit: Effects of Ultrasonic Pretreatment on Drying Time, Energy, Qualitative, and Bioactive Compounds’ Properties[J]. Foods, 2021, 10(5):1006-1006.

49. Igor P T, Aneta W, Krzysztof L, *et al*. Influence of different drying methods on the quality of Japanese quince fruit[J]. LWT, 2019, 114(C):108416-108416.

50. Duan Z Y, Guo Y M, Wang F G. Vacuum freeze-drying rate of fruits and vegetables based on lattice boltzmann method[J]. Editorial Office of Transactions of the Chinese Society of Agricultural Engineering, 2016, 32(14):258-264.

51. Ai Z P, Mowafy S, Liu Y H. Comparative analyses of five drying techniques on drying attributes, physicochemical aspects, and flavor components of Amomum villosum fruits[J]. LWT, 2022, 154.

Reply to all comments point-by-point (Manuscript Number: PONE-D-22-29075)

The authors' replies are in blue.

COMMENTS FOR THE AUTHOR:
Reviewer 1#

1.The findings are not novel and previous studies have already demonstrated that vacuum freeze drying is a better method of drying, but the study has conducted in a very systematic way and has contribution to the mulberry fruit drying.

[Reply] Thank you very much for your comments. Yes, there have been many researches on fruit drying methods, but most of them focus on the influence of drying methods on fruit quality, or the advantages and disadvantages of different drying methods. This paper mainly studies the physical and chemical analysis, nutritional composition, functional composition and antioxidant capacity of fruits under different drying methods. This is one of the reasons for our research. We hope to use mulberry as the test material to get the difference of fruit quality under different drying methods.

2 The introduction of the manuscript is not adequately structured and need more information to be added.

[Reply] The introduction of this article has been added. Thank you for your valuable comments.

3. The material methods are mentioned in detail, but there are some repetitions, which have compromised the quality of the methodology. In addition the authors should write materials and methods comprehensively.

[Reply] Thank you very much for your suggestion. We have rewritten the materials and methods.

4. The data is properly analyzed and have presented in well-illustrated figures, but results need more appropriate description and discussion, the authors should highlight the important findings.

[Reply] We have corrected the part of description and discussion, thank you for your suggestion.

5. In the abstract line 5 to 8 are weird, the authors need to rephrase the sentence.

[Reply] The abstract of this paper has been revised. please see line 12-19 of the "Revised Manuscript with Track Changes", Thank you for your valuable comments.

6. In Introduction line 4, the authors should paraphrase the sentence to give correct sense.

[Reply] Thank you very much for your suggestion. We have corrected it.

7. In Intro line 8 and 9 the sentence making no sense they should be rephrased.

[Reply] Thank you very much for your suggestion. We have corrected it.

8. The format of manuscript should be carefully rechecked to correct mistakes in the format.

[Reply] We have corrected it according to the manuscript format. Thank you for your comments.

9. English must go through a revision, there are some grammar mistakes that compromise the article.

[Reply] We have modified the English language appropriately. Thank you very much for your valuable suggestions. We will continue our efforts to improve our English in the future.

COMMENTS FOR THE AUTHOR:
Reviewer 2#

1.The paper is not written well scientifically (please see below!). The authors should maintain a standard English all through the paper taking care of the grammar.

[Reply] We have modified the English language appropriately. Thank you very much for your valuable suggestions. We will continue our efforts to improve our English in the future.

2. The authors should write a more proper introduction with proper literature review (information) and references.

[Reply] Thank you very much for your suggestion. The introduction part has been revised and appropriate references have been added. please see line 24-65 of the "Revised Manuscript with Track Changes"

3. The authors should mention the logic of using the given 3 cultivars of the mulberry fruit and should mention how they are different among themselves (it must be written in the introduction).

[Reply] Thank you very much for your suggestions. We have revised the manuscript.

In this paper, three mulberry varieties were planted in Dingzhou for 5 years. Bai Yu Wang was a white variety, Longsang was a northern black variety, and Zhong Shen 1 was a southern purple black variety. please see line 62-63 of the “Revised Manuscript with Track Changes”

4. Please write the references for all the information mentioned in the paper e,g ‘’Previous studies on hot air drying and vacuum freeze-drying of mulberry were carried out, but there was a lack of systematic study on the effects of hot air drying and vacuum freeze-drying on the comprehensive quality of different varieties of mulberry…..”
Here the references are missing ??

[Reply] We have added the references for this part, Please see line 421-423 of the “Revised Manuscript with Track Changes”.

5. Please explain the ‘’Methods Section’’ properly and in detail. Vital information related to method is/are missing. E.g., What is the processing time taken of the fruits from the field to the actual processing of the samples (for the experiments)?

[Reply] Thank you very much for your suggestion. We have added this part. Please see line 68-69 of the “Revised Manuscript with Track Changes”.

6. Please write the full name of all the chemical used at the place of its first occurrence in the manuscript and later use the abbreviations e.g ABTS, DPPH, TPTZ. Authors have not mentioned the detail of the chemicals used.

[Reply] We have made some changes in the article, Please see line 71-73 of the “Revised Manuscript with Track Changes”. Thank you very much for your valuable advice.

7. Authors should clarify why they used different ‘’anti-oxidant assays’’ to measure the same thing? Please mention the logic and the benefit if any (also mention references)?

[Reply] References have been reviewed and cited, please refer to lines 489-490 of "Revised Manuscript with Track Changes".

8. In ‘’Materials and Method section’’ the authors have mentioned ‘’Mulberry (Baiyuwang (D1), Longsang (D2) and Zhongshen 1 (D3)) were picked from Dongsheng Ecological Park in Dingzhou City,….’’ The author should use the code D1, D2 and D3 in the abstract itself where they have mentioned the name of the different cultivar of the mulberry. After that the authors should stick to the code (no need to mention full name everywhere!).

[Reply] Thank you very much for your suggestions. We have used codes D1, D2 and D3 in the abstract, and will stick with them for the rest of the article.

9. In the manuscript, the authors have mainly written the results!! They must discuss their results (Discussion part) in proper and a better logical way based on their findings (results) and the known literature in this topic. The results and the discussion part must be well written and should make a better sense.

[Reply] Thank you very much for your suggestions. We have revised the manuscript. Please refer to the manuscript.

10.The ‘’Conclusion Section’’ should also be ‘’re-written’’ properly based on the results with future perspective etc.

[Reply] Thank you very much for your suggestion. We have corrected the content of the conclusion, please refer to the manuscript.

Once again, thank you very much for your comments and suggestions. Those comments are all valuable and very helpful for revising and improving our manuscript.
