# Supplementary material for: Effect of vacuum freeze drying and hot air drying on dried mulberry fruit quality
Source: PLoS One. 2023 Jun 23;18(6):e0283303. doi: 10.1371/journal.pone.0283303 (PMC10289396; doi:10.1371/journal.pone.0283303)
Supplement: Attachment — Submitted filename: Response to Reviewers.docx [file pone.0283303.s002.docx]

**Reply to all comments point-by-point (Manuscript Number: PONE-D-22-29075)**

The authors’ replies are in blue.

**COMMENTS FOR THE AUTHOR:**

**Reviewer 1#**

1.The findings are not novel and previous studies have already demonstrated that vacuum freeze drying is a better method of drying, but the study has conducted in a very systematic way and has contribution to the mulberry fruit drying.

[Reply] Thank you very much for your comments. Yes, there have been many researches on fruit drying methods, but most of them focus on the influence of drying methods on fruit quality, or the advantages and disadvantages of different drying methods. This paper mainly studies the physical and chemical analysis, nutritional composition, functional composition and antioxidant capacity of fruits under different drying methods. This is one of the reasons for our research. We hope to use mulberry as the test material to get the difference of fruit quality under different drying methods.

2 The introduction of the manuscript is not adequately structured and need more information to be added.

[Reply] The introduction of this article has been added. Thank you for your valuable comments.

3. The material methods are mentioned in detail, but there are some repetitions, which have compromised the quality of the methodology. In addition the authors should write materials and methods comprehensively.

[Reply] Thank you very much for your suggestion. We have rewritten the materials and methods.

4. The data is properly analyzed and have presented in well-illustrated figures, but results need more appropriate description and discussion, the authors should highlight the important findings.

[Reply] We have corrected the part of description and discussion, thank you for your suggestion.

5. In the abstract line 5 to 8 are weird, the authors need to rephrase the sentence.

[Reply] The abstract of this paper has been revised. please see line 12-19 of the “Revised Manuscript with Track Changes”, Thank you for your valuable comments.

6. In Introduction line 4, the authors should paraphrase the sentence to give correct sense.

[Reply] Thank you very much for your suggestion. We have corrected it.

7. In Intro line 8 and 9 the sentence making no sense they should be rephrased.

[Reply] Thank you very much for your suggestion. We have corrected it.

8. The format of manuscript should be carefully rechecked to correct mistakes in the format.

[Reply] We have corrected it according to the manuscript format. Thank you for your comments.

9. English must go through a revision, there are some grammar mistakes that compromise the article.

[Reply] We have modified the English language appropriately. Thank you very much for your valuable suggestions. We will continue our efforts to improve our English in the future.

**COMMENTS FOR THE AUTHOR:**

**Reviewer 2#**

1.The paper is not written well scientifically (please see below!). The authors should maintain a standard English all through the paper taking care of the grammar.

[Reply] We have modified the English language appropriately. Thank you very much for your valuable suggestions. We will continue our efforts to improve our English in the future.

2. The authors should write a more proper introduction with proper literature review (information) and references.

[Reply] Thank you very much for your suggestion. The introduction part has been revised and appropriate references have been added. please see line 24-65 of the “Revised Manuscript with Track Changes”

3. The authors should mention the logic of using the given 3 cultivars of the mulberry fruit and should mention how they are different among themselves (it must be written in the introduction).

[Reply] Thank you very much for your suggestions. We have revised the manuscript. In this paper, three mulberry varieties were planted in Dingzhou for 5 years. Bai Yu Wang was a white variety, Longsang was a northern black variety, and Zhong Shen 1 was a southern purple black variety. please see line 62-63 of the “Revised Manuscript with Track Changes”

4. Please write the references for all the information mentioned in the paper e,g ‘’Previous studies on hot air drying and vacuum freeze-drying of mulberry were carried out, but there was a lack of systematic study on the effects of hot air drying and vacuum freeze-drying on the comprehensive quality of different varieties of mulberry…..’’
Here the references are missing ??

[Reply] We have added the references for this part, Please see line 421-423 of the “Revised Manuscript with Track Changes”.

5. Please explain the ‘’Methods Section’’ properly and in detail. Vital information related to method is/are missing. E.g., What is the processing time taken of the fruits from the field to the actual processing of the samples (for the experiments)?

[Reply] Thank you very much for your suggestion. We have added this part. Please see line 68-69 of the “Revised Manuscript with Track Changes”.

6. Please write the full name of all the chemical used at the place of its first occurrence in the manuscript and later use the abbreviations e.g ABTS, DPPH, TPTZ. Authors have not mentioned the detail of the chemicals used.

[Reply] We have made some changes in the article, Please see line 71-73 of the “Revised Manuscript with Track Changes”. Thank you very much for your valuable advice.

7. Authors should clarify why they used different ‘’anti-oxidant assays’’ to measure the same thing? Please mention the logic and the benefit if any (also mention references)?

[Reply] References have been reviewed and cited, please refer to lines 489-490 of "Revised Manuscript with Track Changes".

8. In ‘’Materials and Method section’’ the authors have mentioned ‘’Mulberry (Baiyuwang (D1), Longsang (D2) and Zhongshen 1 (D3)) were picked from Dongsheng Ecological Park in Dingzhou City,….’’ The author should use the code D1, D2 and D3 in the abstract itself where they have mentioned the name of the different cultivar of the mulberry. After that the authors should stick to the code (no need to mention full name everywhere!).

[Reply] Thank you very much for your suggestions. We have used codes D1, D2 and D3 in the abstract, and will stick with them for the rest of the article.

9. In the manuscript, the authors have mainly written the results!! They must discuss their results (Discussion part) in proper and a better logical way based on their findings (results) and the known literature in this topic. The results and the discussion part must be well written and should make a better sense.

[Reply] Thank you very much for your suggestions. We have revised the manuscript. Please refer to the manuscript.

10.The ‘’Conclusion Section’’ should also be ‘’re-written’’ properly based on the results with future perspective etc.

[Reply] Thank you very much for your suggestion. We have corrected the content of the conclusion, please refer to the manuscript.

Once again, thank you very much for your comments and suggestions. Those comments are all valuable and very helpful for revising and improving our manuscript.
